# Supplementary material for: Objective, Quantitative, Data-Driven Assessment of Chemical Probes
Source: Cell Chem Biol. 2018 Feb 15;25(2):194–205.e5. doi: 10.1016/j.chembiol.2017.11.004 (PMC5814752; doi:10.1016/j.chembiol.2017.11.004)

Cell Chemical Biology, Volume *25*

Supplemental Information

Objective, Quantitative, Data-Driven Assessment of Chemical Probes

Albert A. Antolin, Joseph E. Tym, Angeliki Komianou, Ian Collins, Paul Workman, and Bissan Al-Lazikani

Supplemental Information

Objective, Quantitative, Data-Driven Assessment of Chemical Probes

Albert A. Antolin,[1,2] Joe E. Tym,[1] Angeliki Komianou,[1] Ian Collins,[1] Paul Workman[1] & Bissan Al-Lazikani[1,2]

[1]Cancer Research UK Cancer Therapeutics Unit, Division of Cancer Therapeutics and [2]Department of Data Science, The Institute of Cancer Research, London, UK.

Supplemental Figures

Supplementary	Title	Page #
Figure S1	No correlation existing between Information Richness and number of minimum quality chemical tools per each target.	3
Figure S2	Probing activating cancer driver targets.	4
Figure S3	Flow diagram illustrating the logic for the calculation of the scores.	5
Figure S4	Identification and correction of a curation error in ChEMBL affecting chemical probe CCT241533	6
Figure S5	Theoretical examples	7
Figure S6	Most compound-target interactions are not affected by a very broad distribution of affinity values.	8

Figure S1. No correlation existing between Information Richness and number of minimum quality chemical tools per each target, related to Figure 1 and STAR Methods. The red line represents the failed linear regression of the data that has an R^2 of 0.1093.

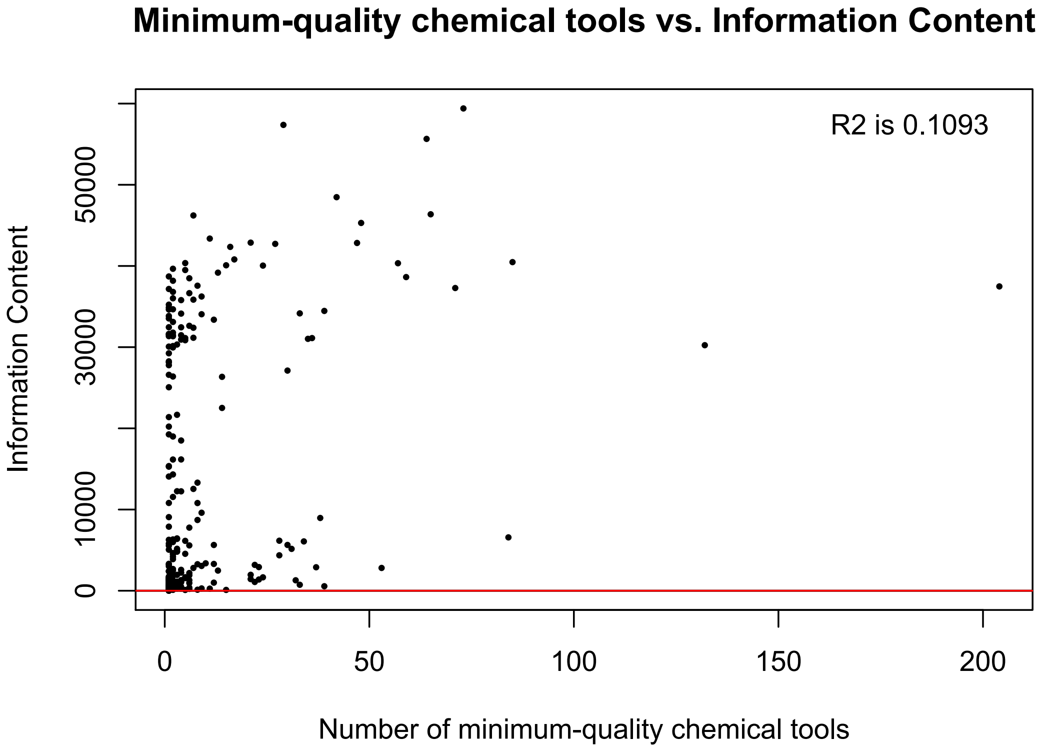

Figure S2. Probing activating cancer driver targets. On the left, pie chart representing the number of cancer driver genes with minimum-quality chemical tools, related to Figure 1. On the right, protein-protein interaction network of the 25 cancer driver genes that have minimum-quality chemical probes obtained from canSAR (Tym et al., 2016). Node size is proportional to the number of compounds tested for the target and shading represents the number of minimum-quality chemical probes, being dark blue the largest number of probes. Edges are coloured depending on the type of protein-protein interaction. Black represents direct binding, cyan represents phosphorylation with arrow showing direction, and magenta represent crystallographycally resolved interactions.

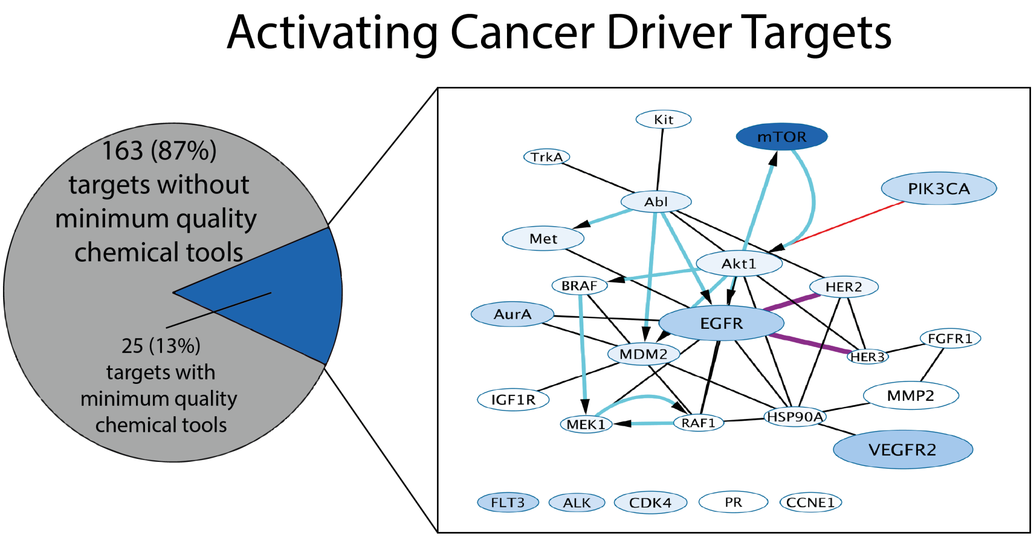

Figure S3. Flow diagram illustrating the logic for the calculation of the scores, related to STAR Methods.

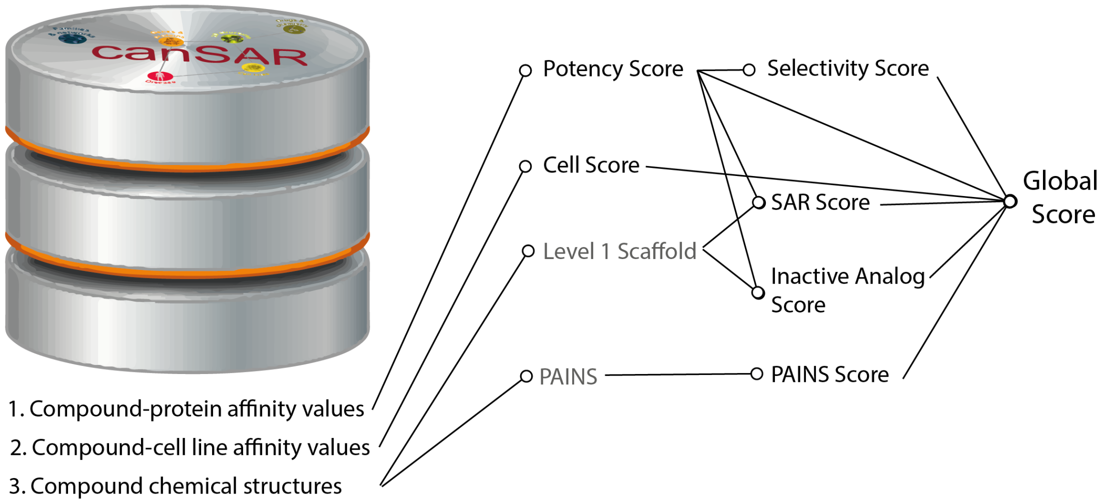

Figure S4. Identification and correction of a curation error in ChEMBL22 affecting chemical probe CCT241533, related to Figure 4. While analysing the reasons for not ranking among the top 20 chemical tools 15 of the chemical probes recommended by The Chemical Probes Portal (Supplementary Table 1-2), we identified that the CHEK2 IC_{50} for CCT241533 had been wrongly curated in ChEMBL22 (CHEMBL1236782) and a value of 30 nM had been given instead of 3 nM as in the original publication. This lower affinity made the probe appear as not 10-fold selective and therefore the Selectivity Score was very low and the Potency Score was also lower than deserved. We have thus corrected this value in canSAR and informed ChEMBL of the error so it can be corrected in future versions of the ChEMBL database. The probe now scores 3rd in our resource when ranking CHEK2 chemical tools by the predefined Global Score. This example illustrates that public databases are not exempt of errors that need to be corrected to make best use of this resource.

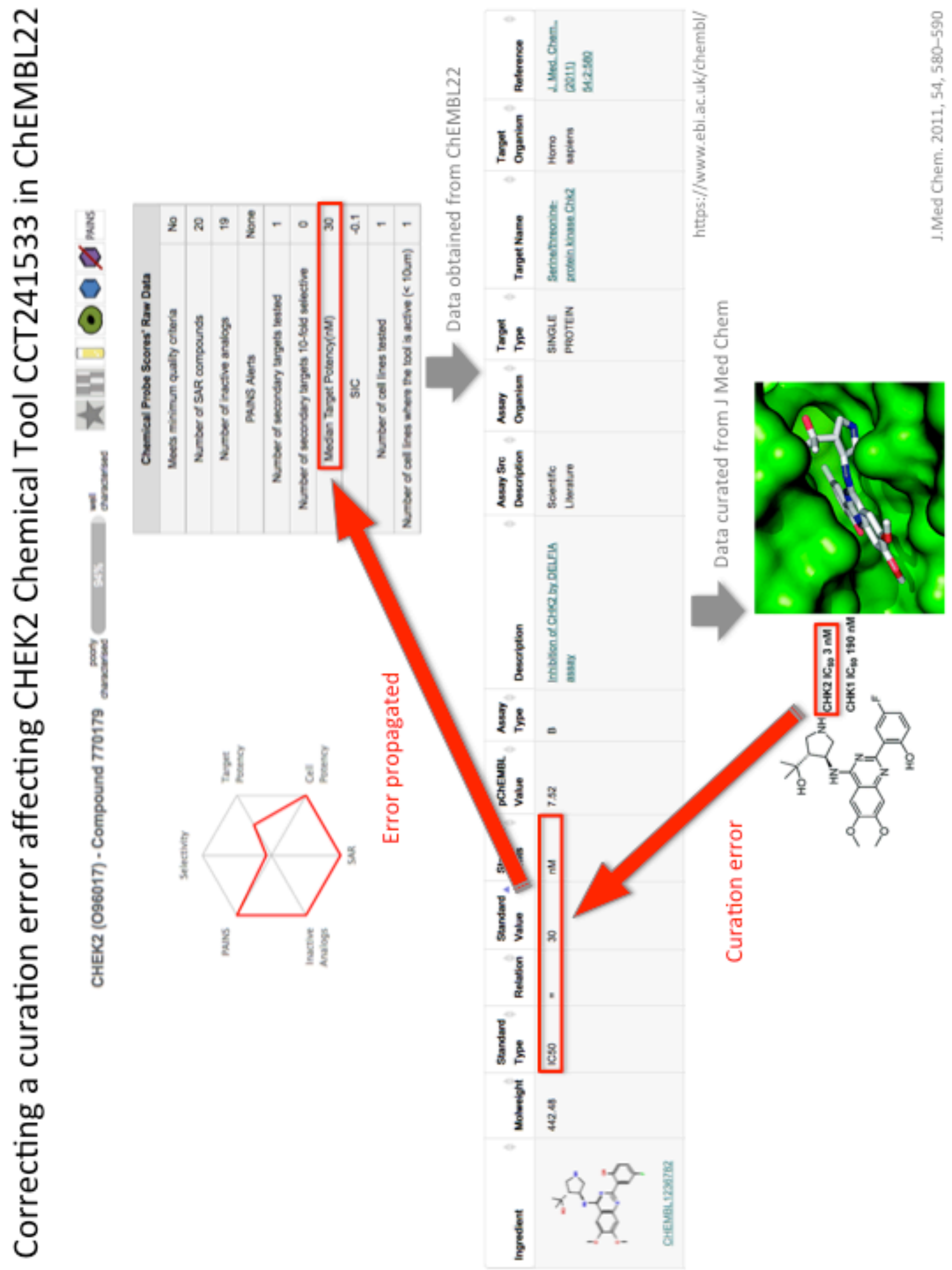

Figure S5. Theoretical examples, related to STAR Methods. (A) Example showing how the differences in selectivity knowledge challenge the comparison of molecules screened against a very different number of targets. (B) Theoretical example illustrating the calculation of the number of off-targets, the differentiation between selective and unselective off-targets and the calculation of first factor of the Selectivity Score. (C) Calculation of the SIC and the Second Factor. (D) Comparing the SIC and Second Factor measures for compounds screened against a very different number of targets.

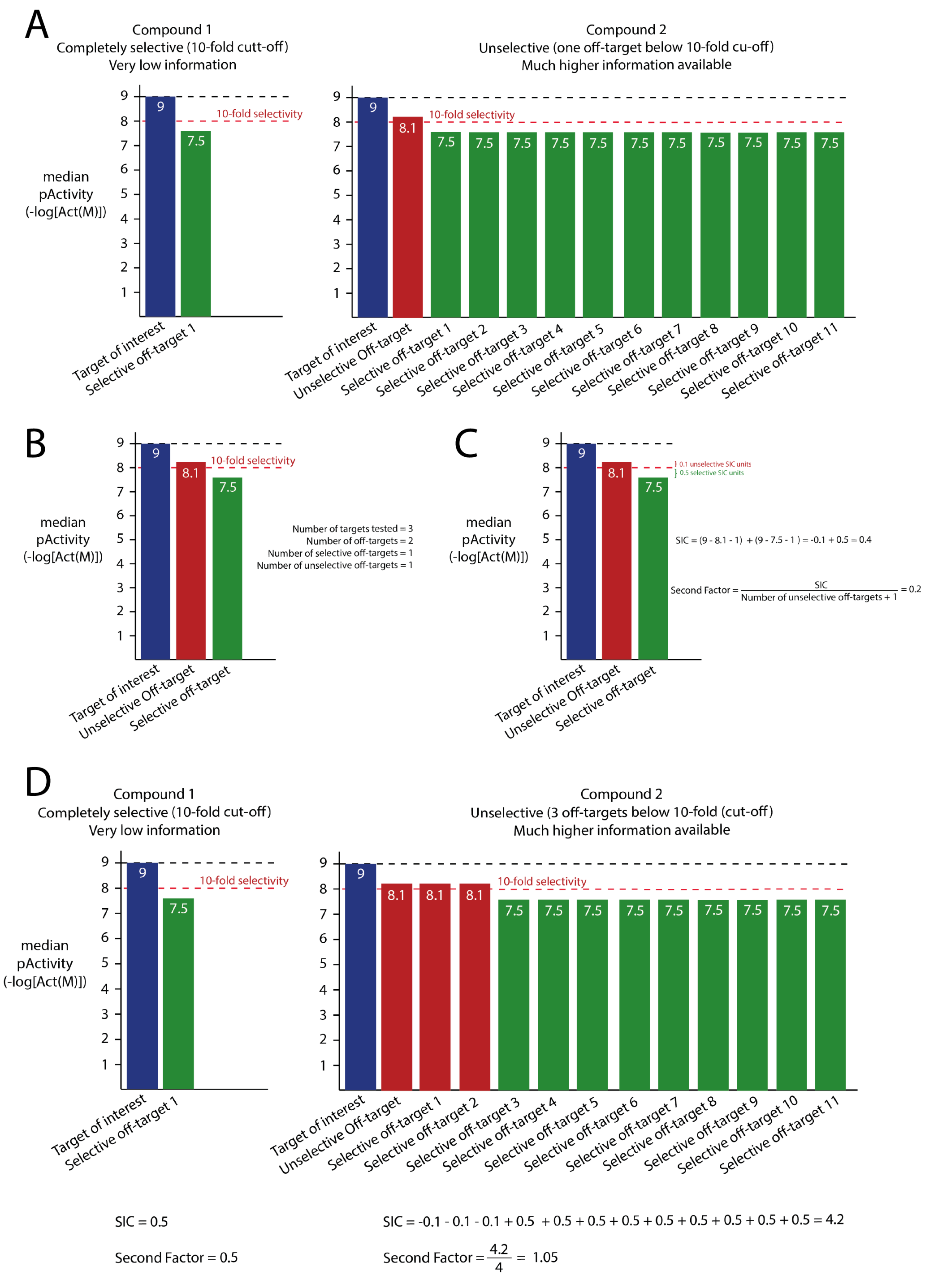

Figure S6. Most compound-target pairs are not affected by a very broad distribution of affinity values, related to STAR Methods. To identify compound-target pairs with broad distribution we calculated the difference between the highest and the lowest values when adding or subtracting the Median Absolute Deviation (MAD) to the Median in a logarithmic scale. As it can be observed in the histogram, for the vast majority of compound-target pairs (96.4%) there is less than 10-fold difference (1 log unit) between these extreme values, thus supporting the use of the median in most cases.

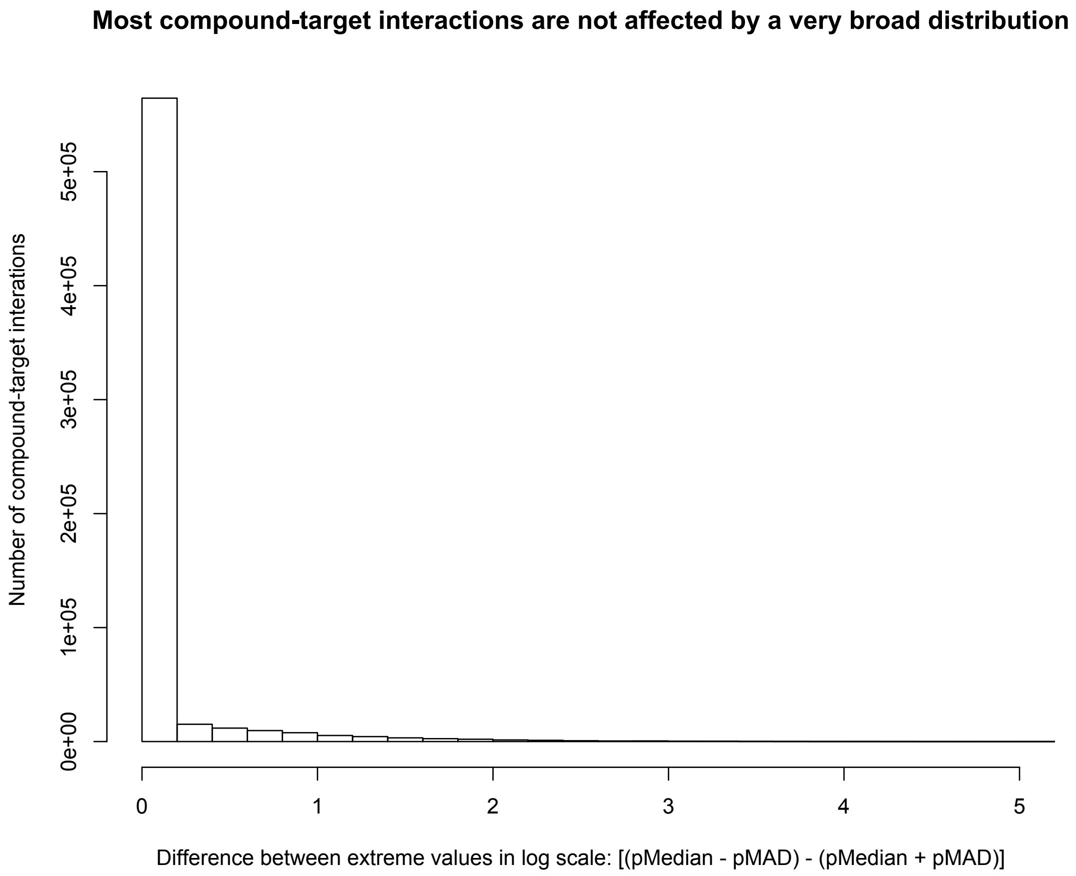

Supplement: Document S1. Figures S1–S6 [file mmc1.pdf]
