# Supplementary material for: Objective, Quantitative, Data-Driven Assessment of Chemical Probes
Source: Cell Chem Biol. 2018 Feb 15;25(2):194–205.e5. doi: 10.1016/j.chembiol.2017.11.004 (PMC5814752; doi:10.1016/j.chembiol.2017.11.004)

Resource

Cell Chemical Biology

# Objective, Quantitative, Data-Driven Assessment of Chemical Probes

## Graphical Abstract

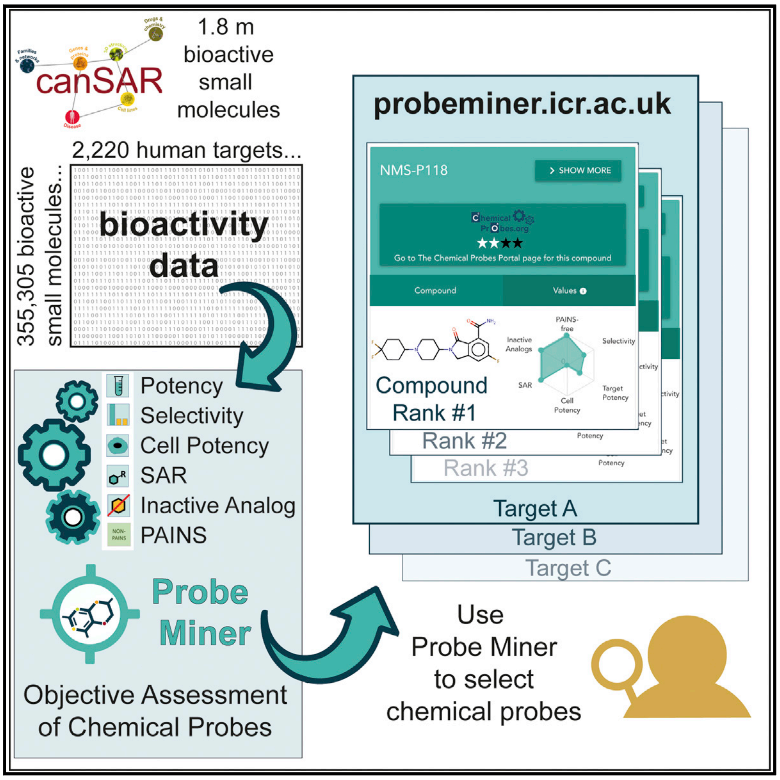


## Authors

Albert A. Antolin, Joseph E. Tym, Angeliki Komianou, Ian Collins, Paul Workman, Bissan Al-Lazikani

## Correspondence

paul.workman@icr.ac.uk (P.W.),
bissan.al-lazikani@icr.ac.uk (B.A.-L.)

## In Brief

Chemical probe selection remains largely subjective, not utilizing extensive medicinal chemistry data, causing widespread use of flawed probes. Antolin et al. describe the Probe Miner resource, empowering objective, quantitative, data-driven assessment of chemical probes based on regularly updated large-scale data.

## Highlights

- Systematic analysis of chemical probes uncovers new insights and limitations
- Novel data-driven scoring enables objective assessment of chemical probes
- A public, regularly updated resource equips researchers to evaluate probes
- Used alongside expert curation, provides a powerful resource for probe selection

Antolin et al., 2018, Cell Chemical Biology *25*, 194–205
February 15, 2018 © 2017 The Authors. Published by Elsevier Ltd.
https://doi.org/10.1016/j.chembiol.2017.11.004

# Objective, Quantitative, Data-Driven Assessment of Chemical Probes

Albert A. Antolin,[1,2] Joseph E. Tym,[2] Angeliki Komianou,[2] Ian Collins,[1] Paul Workman,[1,*] and Bissan Al-Lazikani[1,2,3,*]
[1]Cancer Research UK Cancer Therapeutics Unit, Division of Cancer Therapeutics
[2]Department of Data Science
The Institute of Cancer Research, 15 Cotswold Road, London SM2 5NG, UK
[3]Lead Contact
*Correspondence: paul.workman@icr.ac.uk (P.W.), bissan.al-lazikani@icr.ac.uk (B.A.-L.)
https://doi.org/10.1016/j.chembiol.2017.11.004

## SUMMARY

**Chemical probes are essential tools for understanding biological systems and for target validation, yet selecting probes for biomedical research is rarely based on objective assessment of all potential compounds. Here, we describe the Probe Miner: Chemical Probes Objective Assessment resource, capitalizing on the plethora of public medicinal chemistry data to empower quantitative, objective, data-driven evaluation of chemical probes. We assess >1.8 million compounds for their suitability as chemical tools against 2,220 human targets and dissect the biases and limitations encountered. Probe Miner represents a valuable resource to aid the identification of potential chemical probes, particularly when used alongside expert curation.**

## INTRODUCTION

Small-molecule chemical probes are important tools for exploring biological mechanisms and play a key role in target validation (Blagg and Workman, 2017; Bunnage et al., 2013; Frye, 2010; Workman and Collins, 2010). However, selection of chemical probes is largely subjective and prone to historical and commercial biases (Arrowsmith et al., 2015; Workman and Collins, 2010). Despite many publications discussing the properties of chemical probes and the proposal of "fitness factors" to be considered when assessing chemical tools, scientists commonly select probes through web-based searches or previous literature sources that are heavily biased toward older and often flawed probes, or use vendor catalogs that do not discriminate between probes (Arrowsmith et al., 2015; Blagg and Workman, 2017; Workman and Collins, 2010).

The Chemical Probes Portal (Arrowsmith et al., 2015; http://www.chemicalprobes.org) has been launched as a public, non-profit, expert-driven chemical probe recommendation platform, and this emerging resource is already contributing to improved chemical probe selection (Blagg and Workman, 2017). However, expert curation, by definition, can be limited in its coverage and would benefit from a complementary, frequently updated, systematic, data-driven, objective, and comprehensive approach that enables researchers to keep track of the fast-moving advances in chemical biology-relevant data at a scale difficult to reach with expert curation, allowing unbiased comparison of the quality of large numbers of probes. Recently, a scoring system to prioritize chemical tools for phenotypic screening based on expert weighting of public and highly curated private databases was described (Wang et al., 2016). However, such resources are not available to the majority of translational researchers. A public resource that democratizes comprehensive data-driven chemical probe assessment is still lacking and would greatly contribute to target validation and mechanistic studies performed outside industry. Here, we analyze at scale the scope and quality of published bioactive molecules and uncover large biases and limitations of chemical tools and their representation in public databases. We provide the online Probe Miner: Chemical Probes Objective Assessment resource where we integrate large-scale public data to enable objective, quantitative, and systematic assessment of chemical probes.

## RESULTS

### Probing the Liganded Proteome Using Public Databases

An ambitious early grand challenge of chemical biology was to identify a chemical tool for each human protein (Schreiber, 2005; Workman and Collins, 2010). To assess the level of progress toward meeting this challenge, we first defined the set of 20,171 curated, validated human proteins in Uniprot (The Uniprot Consortium, 2017). We then utilized the canSAR knowledgebase integration (Tym et al., 2016; http://cansar.icr.ac.uk) of major, curated, public medicinal chemistry data (including ChEMBL and BindingDB, see STAR Methods) to determine the fraction of these proteins that are known to interact with small-molecule compounds (The Uniprot Consortium, 2017; Tym et al., 2016). We find that only 11% (2,220 proteins) of the human proteome has been liganded (Figure 1A). This percentage is still very low even if we compare it with the 22%–40% of the proteome that is estimated to be potentially druggable (Figure 1A; Bulusu et al., 2014; Finan et al., 2017; Tym et al., 2016).

To be effective tools for mechanistic biological experiments and target validation, chemical probes must satisfy at least some basic criteria for the key properties (fitness factors) of potency, selectivity, and permeability (Workman and Collins, 2010). To assess how many of the compounds available in public databases would be useful in this context, we establish key minimal

 © 2017 The Authors. Published by Elsevier Ltd.
This is an open access article under the CC BY license (http://creativecommons.org/licenses/by/4.0/).

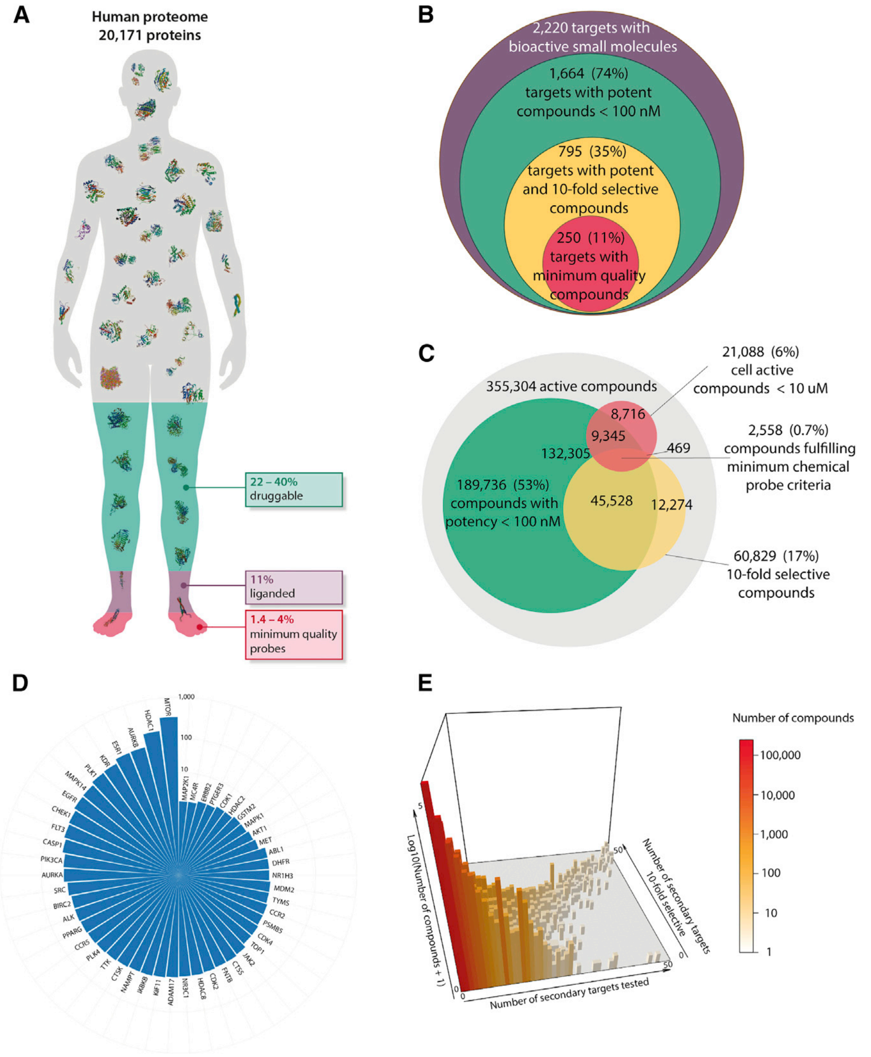


**Figure 1. Global Analysis of Chemical Probes as Described in Public Databases Uncovers Major Limitations and Biases**

(A) Infographic showing a human silhouette representing the human proteome and areas indicating: the proportion of the proteome estimated to be druggable but currently unliganded (green; Finan et al., 2017; Tym et al., 2016); the proportion found to have been already liganded (purple; see STAR Methods); and the proportion that can be studied currently with chemical tools fulfilling minimum requirements of potency, selectivity, and permeability (red; see STAR Methods).

(B) Venn diagram illustrating the proportion of the 2,220 liganded human protein targets that can be studied with chemical tools fulfilling minimum requirements of potency, selectivity, and permeability.

(C) Venn diagram illustrating the number of chemical compounds fulfilling minimum requirements of potency, selectivity, and permeability.

(D) Top 50 targets with the largest number of compounds fulfilling minimum requirements of potency, selectivity, and permeability.

(E) Compound selectivity per number of targets tested uncovers a very reduced exploration of compound selectivity.

See also Figures S1 and S2.

criteria that should be satisfied: (1) potency: 100 nM or better on-target biochemical activity or binding potency; (2) selectivity: at least 10-fold selectivity against other tested targets; and (3) permeability: as no large-scale experimental measures of permeability are available, we use reported activity in cells (independent of the target and wherever available in our sources) as a proxy and set a minimum concentration requirement of 10 $\mu$M (see STAR Methods). It is important to stress that these three minimal requirement levels do not guarantee that a chemical tool would be suitable for biological investigation, but all suitable tools should in principle meet these basic requirements.

From the >1.8 million total compounds (TC) available in public databases, we find that only 355,305 human active compounds (HAC) have some acceptable level of biochemical activity (<10 $\mu$M; see STAR Methods) reported against a human protein. Of these, 189,736 (10.5% TC, 53% HAC) have measured biochemical activity or binding potency of 100 nM or better. However, when considering selectivity, we find that only 93,930 compounds have reported binding or activity measurements against two or more targets. Of these, only 48,086 (2.7% TC, 14% HAC) satisfy both our minimal potency and selectivity criteria (Figure 1C). Thus, exploration of compound selectivity in the medicinal chemistry literature appears alarmingly limited (Figure 1E). Moreover, we find that the compounds that satisfy our minimal potency and selectivity criteria allow the research community to probe only 795 human proteins (4% of the human proteome) and at best 18% of the estimated druggable proteome (Figures 1A–1C). Finally, when additionally considering cellular potency of 10 $\mu$M or better, we find that the number of minimal quality probes is reduced even further to 2,558 (0.7% HAC). Under these combined criteria, based on the information available in public medicinal chemistry databases, compounds fulfilling minimum requirements would allow the research community to probe with real confidence only 250 human proteins (Figure 1B). This represents an unacceptably low percentage (1.2%) of the human proteome.

The amount of information available for a given protein target will clearly have an impact on any statistical analysis of its corresponding chemical tools. To assess the role of differing levels of experimental characterization, we define the "Information Richness" as follows: for each target, A, we collect all small molecules (C) shown to be active against this target. For each compound, we then count the number of targets (T) against which it has been tested, regardless of activity level. Thus, the Information Richness, $IR_A = \sum_{C=1}^{C=n} T$ (see STAR Methods for details).

As expected, we find large biases in the amount of data in public medicinal chemistry databases available for different protein targets. We also observe a wide range in the number of

compounds fulfilling our minimum criteria across all the protein targets (0–204; Figure 1D). For example, some targets have many well-characterized compounds, several of which fulfill our minimum criteria; e.g., the metalloprotease ADAM17 has 1,433 active compounds of which 31 satisfy our minimal criteria. Other protein targets have large numbers of compounds with differing degrees of characterization, yet few, if any, satisfy our minimal criteria; e.g., JAK1 has 1,560 active compounds, none of which satisfy our minimal criteria with the data available (Figures 1D and S1).

Several factors could influence the observed biases, for example, the availability of selective probes varies significantly across the analyzed protein targets (0–896 selective compounds). The identification of selective probes may be simpler for some targets that have distinctive binding sites (e.g., PPAR$\gamma$) and difficult for others that share closely similar binding sites with numerous family members (e.g., ABL1). Increasing the public availability of large-scale panel screens for many compounds against many targets will certainly help expand the information matrix required to identify good quality probes. Indeed, half of the 50 protein targets with the greatest number of minimum-quality probes are kinases, which frequently benefit from broad kinome selectivity screens and researchers' and peer reviewers' awareness that selectivity is a critical issue in this target class (Figure 1D). However, this brute-force selectivity profiling approach alone is insufficient. Overall, we find poor correlation ($R^2$ = 0.1) between the number of reported experimental measurements and the number of minimum-quality probes (Figure S1). This finding indicates that our community needs to be smarter in designing and testing compounds, for example, optimizing ligand efficiency for probes based on both molecular weight and lipophilicity may inherently improve selectivity (Hopkins et al., 2014), in addition to increasing the throughput of data generation.

## Probing Disease Genes

Our systematic approach allows us to investigate, more globally, how well existing chemical tools equip us to probe mechanistically the function of disease genes, which is particularly important for therapeutic target validation. As an exemplar, we analyze data for a set of 188 cancer driver genes (CDG) with activating genetic alterations (Rubio-Perez et al., 2015) and examine the availability of minimal quality chemical probes for these drivers. We find that 73 (39% CDG) have already been liganded, and of these 25 (13% CDG) have chemical tools in public databases fulfilling minimum requirements of potency, selectivity, and permeability (Table S1, Figure S2). This is a significantly higher fraction than we find across the proteome as a whole (1.2% as described earlier; Figure 1B). The reason for this elevated fraction is that the CDGs contain many long-established disease genes that have been heavily investigated for chemical ligands. Nevertheless, 87% of CDG do not have a minimum-quality chemical tool (Table S1). Moreover, the vast majority of chemical tools concentrate on relatively few protein targets, further demonstrating the documented trend to focus research efforts in areas of science that are already well studied (Table S1, Figure S2; Edwards et al., 2011; Fedorov et al., 2010). This analysis further uncovers a severe lack of chemical probe availability and significant bias where tools are available.

## Objective Assessment of Chemical Probes

Given the biases and limitations discussed above, it is imperative that researchers can comprehensively access all the data publicly available to facilitate objective and data-driven analysis. Importantly, this will help enable them to select the best characterized chemical probes available for their protein target of interest, and also allow them to understand probe liabilities and limitations at the outset. To this end, we describe a scoring metric that utilizes >3.9 million bioactivity data points publicly available in canSAR (Tym et al., 2016) to enable rational prioritization of chemical probes.

To create a metric that allows objective, data-driven ranking of all compounds tested for a particular protein target, we developed a set of six scores mirroring our previously described fitness factors (Workman and Collins, 2010). Namely, Potency Score, Selectivity Score, Cell Score, Structure-Activity Relationship (SAR) Score, Inactive Analog Score, and PAINS Score (see STAR Methods; Figure S3). For ease of use, we predefine a default weighting of these scores, which emphasizes the importance of potency and selectivity (see STAR Methods). However, in addition, we also provide the facility for researchers to adapt and customize the weights to suit their own questions, expertise, and preferences.

Using our default scoring scheme allows us to highlight compounds that make good candidates for chemical probes and defines their key limitations. For example, we assessed 1,346 compounds for the class I phosphatidylinositol 3-kinase (PI3K), PIK3CB. The five highest-ranking probes include the clinical candidate pictilisib or GDC-0941 (top rank; Folkes et al., 2008) and the frequently used probe PI-103 (second ranked; Raynaud et al., 2007; Figure 2), both of which have been widely profiled in large kinome panels. However, our assessment shows that both these compounds have certain selectivity liabilities due to cross-PI3K activity (Figure 2). The PIK3CB/PIK3CD inhibitor AZD-6482 ranks tenth (Figure 2), due to its partial PI3K selectivity toward PIK3CA and PIK3G (Andrs et al., 2015), and most other PIK3CB-selective chemical series are also represented among the top scoring probes (Andrs et al., 2015). It is worth highlighting that Probe Miner can also be useful in disincentivizing the use of low-quality or flawed chemical probes that continue to pollute the chemical biology literature (Arrowsmith et al., 2015). An example is LY294002, which is still widely used as a chemical tool inhibitor for PI3K despite the fact that its weak potency and lack of selectivity have been widely communicated in reviews (Arrowsmith et al., 2015; Blagg and Workman, 2014). LY294002 ranks as 63$^{rd}$ for PIK3CB in Probe Miner, and we hope that its low ranking by objective assessment will further discourage the use of this historical but promiscuous compound as a probe for PI3Ks.

Furthermore, our systematic assessment of potential PIK3CB probes additionally highlights another set of interesting compounds with properties exemplified by canSAR1019166 (Sanchez et al., 2012). This ranks third using our default scoring (Figure 2) and is both potent and, unlike pictilisib and PI-103, more selective for PIK3CB versus other PI3K proteins. Since no reports of screening canSAR1019166 against wider kinase panels are in the public domain as yet, other selectivity liabilities may emerge in future. In addition, this compound may not be readily available commercially. There are also potentially important compounds whose broader biochemical characterization is

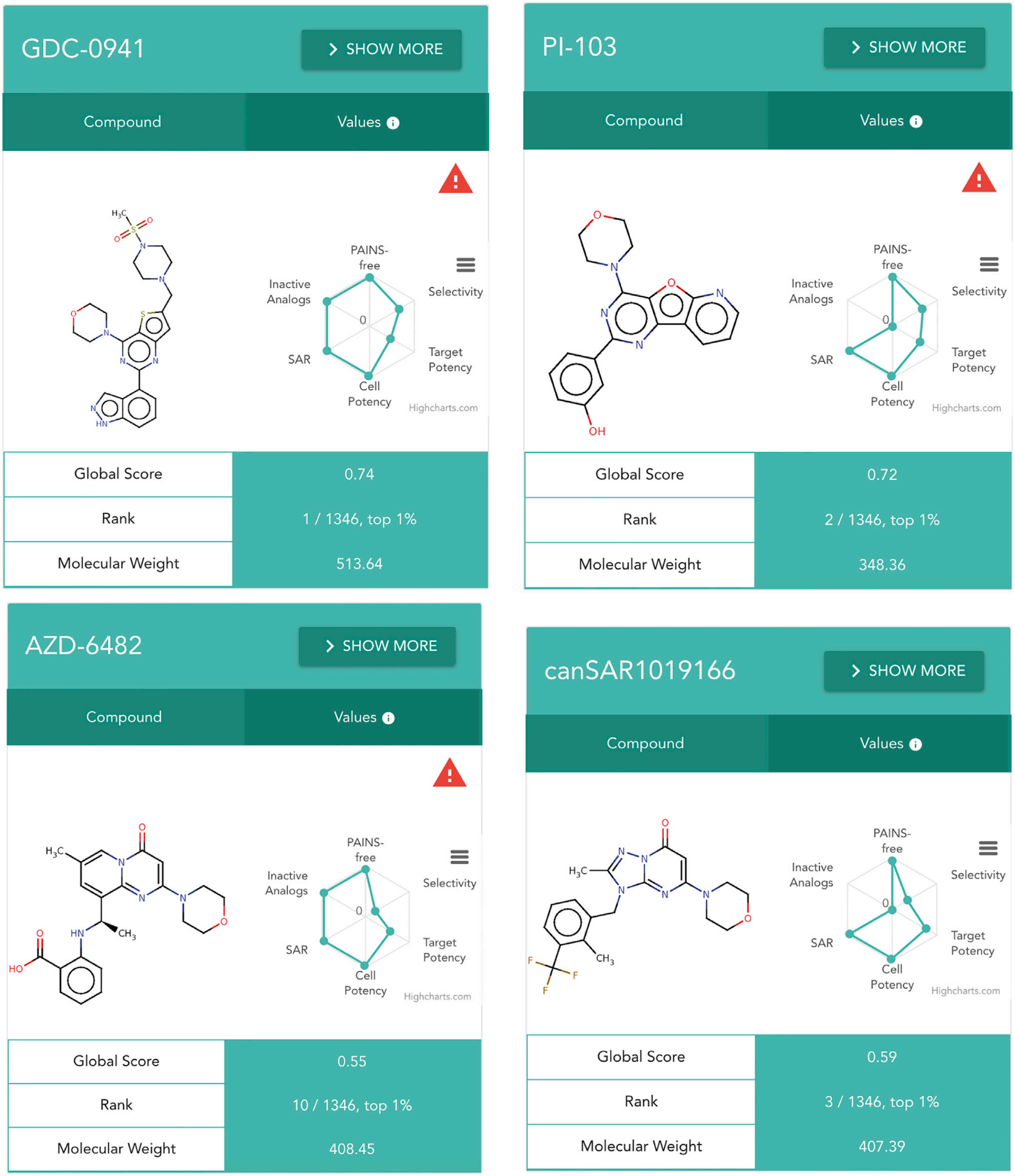


**Figure 2. Chemical Probe Cards for Highest-Ranked PIK3CB Compounds in Probe Miner, Comprising the Chemical Structure and the Radar Plot with the Corresponding Chemical Probe Scores**

Where probes have also been curated by the Chemical Probes Portal, their expert review star rating is also displayed. Moreover, when a compound is not 10-fold selective against another protein, a danger icon (red triangle) is shown to alert the researcher that there might be selectivity liabilities when using those compounds as PIK3B chemical probes.

not captured in public medicinal chemistry databases, and it is thus not possible to appropriately assess them using our unbiased approach. For example, this is the case for the PIK3CB-selective clinical candidate GSK-2636771 (Andrs et al., 2015; Mateo et al., 2017), which is currently not highly ranked in our resource.

## Probe Miner: a Public Resource for Objective Assessment of Probes

To empower the community to utilize the data-driven approach, we have created Probe Miner and have made it publicly available (http://probeminer.icr.ac.uk; Figure 3). This is a user-friendly, interactive web-based resource that allows researchers access to the probe data and probe rankings for the selected protein target, as well as full customization of the scoring criteria and the ability to deep-dive into the data. We will maintain Probe Miner and provide automatic updates following the release of new versions of the public databases that are integrated to ensure topicality.

Probe Miner is a target-centric, systematic probe assessment resource. Accordingly, Probe Miner is designed to be searched by target. After selection of the desired protein target, we provide an interactive graphical overview page (Figure 3 and video tutorial in Movie S1); note that Figure 3 shows the large-screen version but the website automatically adapts to multiple devices and screen sizes. The overview page comprises three major sections as follows: (A) Summaries of the data and statistical analyses using our algorithm, including colored icons (Table 1) that provide immediate visual indication of the overall quality and known or potential liabilities of compounds for the selected target; (B) easy-to-navigate distribution of the 20 highest-ranking probes, as well as tools to customize the scores, weights, and ordering of probes; (C) a compound viewer interactively linked to the distribution, which shows the chemical structure and key information for the probe, as well as the values of the six score components as a radar plot. As Probe Miner is intended to complement expert curation in the Chemical Probes Portal, we highlight the compounds in our resource that are also assessed in the Portal and provide direct links to their individual pages at the Portal site. Movie S1 provides a detailed tutorial on how to navigate the resource.

It is important to stress that the selection of the “best” probes must always be tailored to the scientific question under investigation and, therefore, the final decision on which tools to select must always be undertaken by the individual researcher. We provide our predefined default weightings for calculating the Global Score, which users may prefer for speed and convenience. However, for researchers who wish to modify ranking criteria and score components, we provide a “Settings” panel, which enables advanced options (Figure 3D and Movie S1; see STAR Methods). Here, researchers can set the weights of each of the individual fitness factor scores that contribute to the overall Global Score so that these can be adapted to individual user needs. Through the Settings panel (Figure 3D), researchers can also customize the display and also the ordering of probes (e.g., according to potency or selectivity) as required.

From the Probe Miner overview page for a given protein target, researchers can navigate easily to individual probe pages (Figure 3E and Movie S1). These synopsis pages provide details of the chemical structure; physicochemical properties; and cross-references to key public resources, including canSAR, ChEMBL, BindingDB, the PDB, and Chemical Probes Portal; as well as also indicating synonyms for probes and commercial availability (Figure 3E and Movie S1). The raw data required to generate the scores for the given probe are accessible here in a tabular format, together with the radar plot displaying the various scores (from 0 to 1 with 1 being the highest rating) in addition to the compound’s Probe Miner rankings. The full protein activity profile (the reported activity of the compound against all tested proteins as contained in canSAR) is also provided as a bar plot displaying the median biochemical activities or binding affinities on a logarithmic scale (e.g., $pIC_{50}$ or $pK_d$) for the compound. This enables a quick and easy overview of the selectivity of the compound for the target of interest.

To view all chemical probes for a given protein target, a Chemical Probes Table page in Probe Miner provides tabular access to all the assessed compounds for that target, together with the complete corresponding raw data. This facilitates filtering and allows full download of all the data to enable the chemical biology community to further develop assessment and prioritization methodologies (Movie S1).

The power of our Probe Miner resource is the objective, systematic, regularly updated assessment that relies on public medicinal chemistry databases. However, as illustrated throughout our analysis, the inevitable limitations in data availability or curation can pose a significant challenge in some cases. We believe that arming researchers with all the available information and highlighting potential areas of error or bias is key to empowering them to make the best-informed decisions. Since selectivity is a particular concern with chemical probes (Arrowsmith et al., 2015; Blagg and Workman, 2017), in order to alert researchers to cases where this may be a problem, we have incorporated a red triangular “danger” icon that warns researchers when a chemical probe appears to fail the criterion of 10-fold selectivity against another protein based on the data available to the resource. The easy access we provide to the full protein activity profile at the respective chemical probe page enables a quick visual impression of the assessed selectivity of each chemical tool for the target of interest (Movie S1) while links to the expert-curated Chemical Probes Portal are provided to draw attention to probes recommended by experts. Moreover, our objective assessment performed at scale in Probe Miner can identify compounds that we rank as good potential probe candidates, but which are not currently curated in the Chemical Probes Portal so that they can be considered for evaluation at the Portal. We also highlight potential probes that are not commercially available so that vendors can consider them for inclusion in their catalogs.

To help address errors and inaccuracies in public databases, we carry out continuous curation of the data underlying our Probe Miner resource and have established an email address (chemprobes@icr.ac.uk) through which we can be contacted by any researcher who identifies such errors or inaccuracies affecting the objective assessment of chemical probes. Even high-quality public databases are not exempt from errors and inaccuracies that are extremely challenging to identify and fix. Data-driven approaches rely on the quality of the data they use, and it is thus paramount that we as a community address

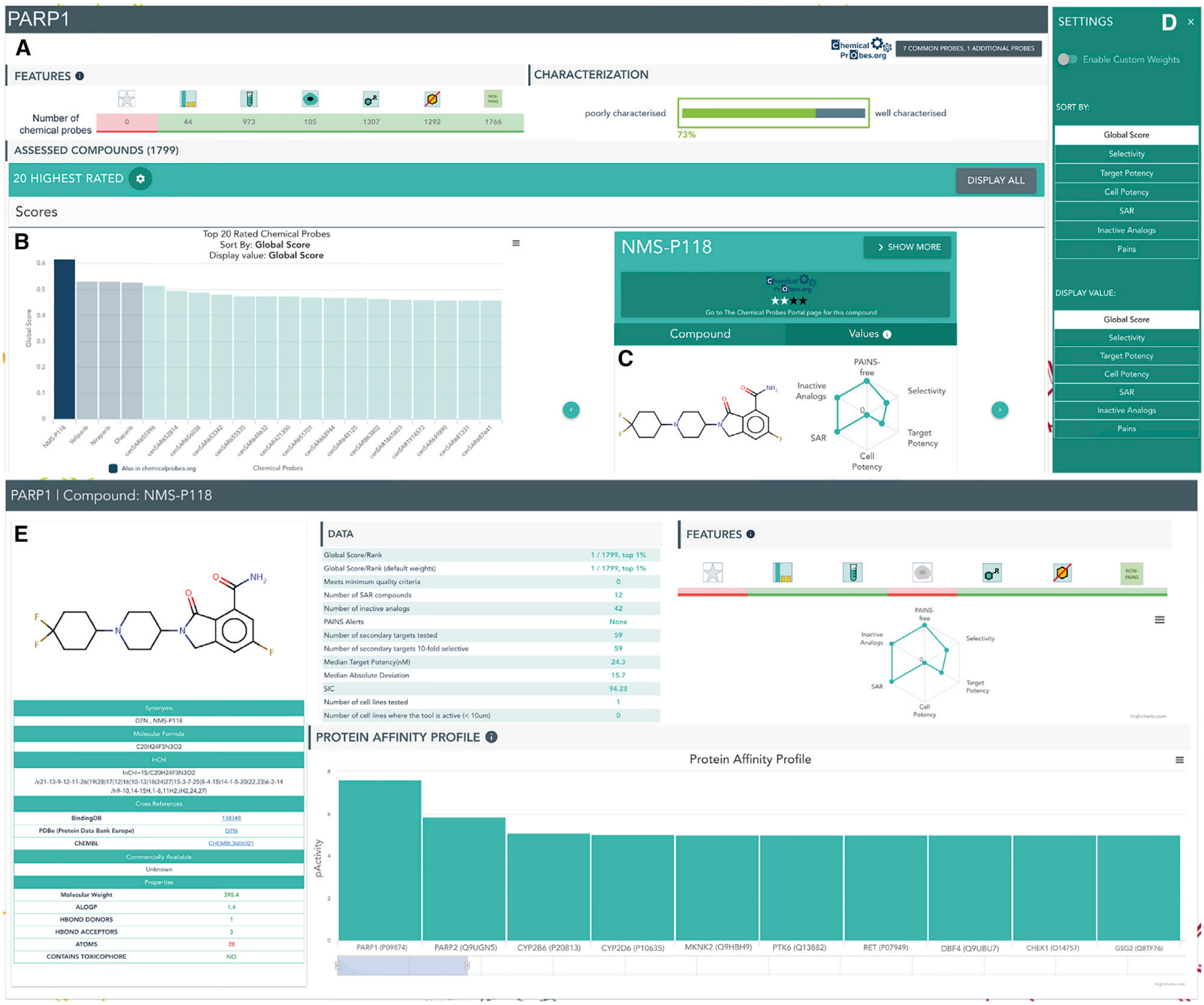


**Figure 3. Probe Miner Resource**

Snapshot of the overview and chemical tool pages of the resource using the human PARP1 protein as an example.

(A) Summaries of the data and statistical analyses using our algorithm. Colored icons provide immediate visual indication of the overall quality and liabilities of compounds for this target, and a link to the Chemical Probes Portal is provided when this target has expert-curated compounds in the Portal.

(B) Easy-to-navigate distribution of the 20 top-ranking probes.

(C) A compound viewer interactively linked to the distribution, which shows the chemical structure and key information for the probe, as well as the values of the six score components as a radar plot. Compounds that are also expert-curated by the Chemical Probes Portal are highlighted in blue and links to the Portal are also provided.

(D) Easy-to-navigate settings panel to enable customization of the Global Score, displays, and rankings.

(E) Individual chemical probe pages where detailed information is provided, including links to other resources, commercial availability, raw data to generate the scores, and a target profile to provide an overview of compound selectivity. To learn more about how to use and navigate Probe Miner, we prepared a video tutorial (See Movie S1).

the errors and inaccuracies in public databases in order to maximize the benefit derived from them.

## Comparing Probe Miner and the Chemical Probes Portal: Complementary and Synergistic Resources

Using our predefined Global Score, we compare the top-ranked chemical probes in Probe Miner with the expert-curated probes available in the Chemical Probes Portal (Arrowsmith et al., 2015). For this analysis, we focus on the selective probes that are curated by the Chemical Probes Portal and that are assigned to no more than two targets within the Portal (data collected on February 6, 2017; see Figure 4 and STAR Methods). Of the 133 probes in the Chemical Probes Portal on that date, 71 were associated with no more than 2 targets and recommended by experts (rating $\geq 3$; see STAR Methods). We find that 46 of these 71 probes, corresponding to 45 targets, could be mapped to public

**Table 1. Chemical Probe Score Icons**

| Icon Name | Description | Image |
|---|---|---|
| Target Selectivity | Denoted by a histogram icon, it shows whether a compound inhibiting this protein is screened against at least one other target and has at least 10-fold selectivity against any other target | 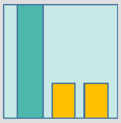 |
| Target Potency | Denoted by a test tube icon, it shows whether a compound inhibits this target with at least 100 nM potency | 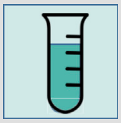 |
| Cell Potency | Denoted by a cell, it shows whether a compound binding to the target of interest is active in a cell line with at least 10 μM potency | 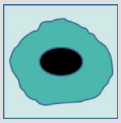 |
| Minimum Standard | Denoted by a star, it is an aggregate of the three previous scores (which themselves are independent from each other), indicating whether there are compounds inhibiting this target with minimum standards of target potency (pActivity $\geq$7), selectivity (at least one tested off-target and 10-fold selectivity against off-targets) and cell potency (activity below 10 μM in at least one cell line) simultaneously. It is a key icon showing whether a compound fulfilling these minimum-quality requirements is found in publicly available databases | 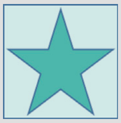 |
| SAR | Denoted by a benzene ring with an 'R' group, it indicates that there is at least one compound binding to this target that has SAR as defined by the SAR Score (see above) | 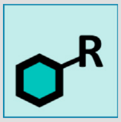  |
| Inactive Analog | Denoted by a barred benzene ring, it indicates that there is at least one inactive analog of the compound as defined by the Inactive Analog Score (see above) | 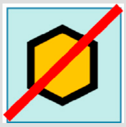 |
| PAINS | Denoted by a NON-PAINS icon, it shows that there is at least one compound inhibiting this target that has no PAINS alerts as defined in the PAINS Score | NON-PAINS |

The chemical probe scores have been adapted to a binary representation in order to facilitate a quick and intuitive evaluation of chemical probe quality. Seven icons illustrate the six chemical probe scores and the minimum standard (see STAR Methods) and can be shown in color or in gray scale depending on the chemical tool fulfilling the description criteria.

databases. Using Probe Miner's preset weightings for the Global Score, 31 (67%) of the selected 46 Portal probes rank in the top 20 in Probe Miner, and 18 (39%) rank among the top 5 (Figure 4 and Table S2). Our analysis of the 15 expert-recommended probes that fail to reach high rankings uncovers the incompleteness of data available in public databases (often because the probe was published in a non-indexed journal) and also the inaccuracy of public data are the major limitations (Tables S2 and S3). As the purpose of our resource is to complement the Chemical Probes Portal with strictly objective large-scale, data-driven information, we explicitly exclude any curated probes that have no data in the underlying medicinal chemistry databases. However, as mentioned, we do provide a link to the Chemical Probes Portal in the features section of the target overview page to alert researchers when a target has probes in the Portal that might not be present in public medicinal chemistry databases. Moreover, to address the broader lack of coverage of chemical biology data in public medicinal chemistry databases, we are actively expanding the canSAR knowledgebase to curate key missing literature. In future, this growing knowledgebase will further enhance our objective assessment and increase the overlap between our resource and the Chemical Probes Portal, which is itself also being extended through ongoing inclusion of additional probes.

Importantly, our analysis highlights 193 compounds with high rankings (in the top 5) in Probe Miner that are not yet curated within the Chemical Probes Portal and that may complement the tools recommended by the Portal to explore the corresponding protein targets of interest. This again highlights the clear synergy of combining the large-scale objective assessment of all available compounds with in-depth but only partially complete coverage of expert curation. To maximize this synergy, we are now collaborating with the Chemical Probes Portal to share information and, for example, to recommend probes identified by our objective assessment method for expert curation at the Portal (see example below).

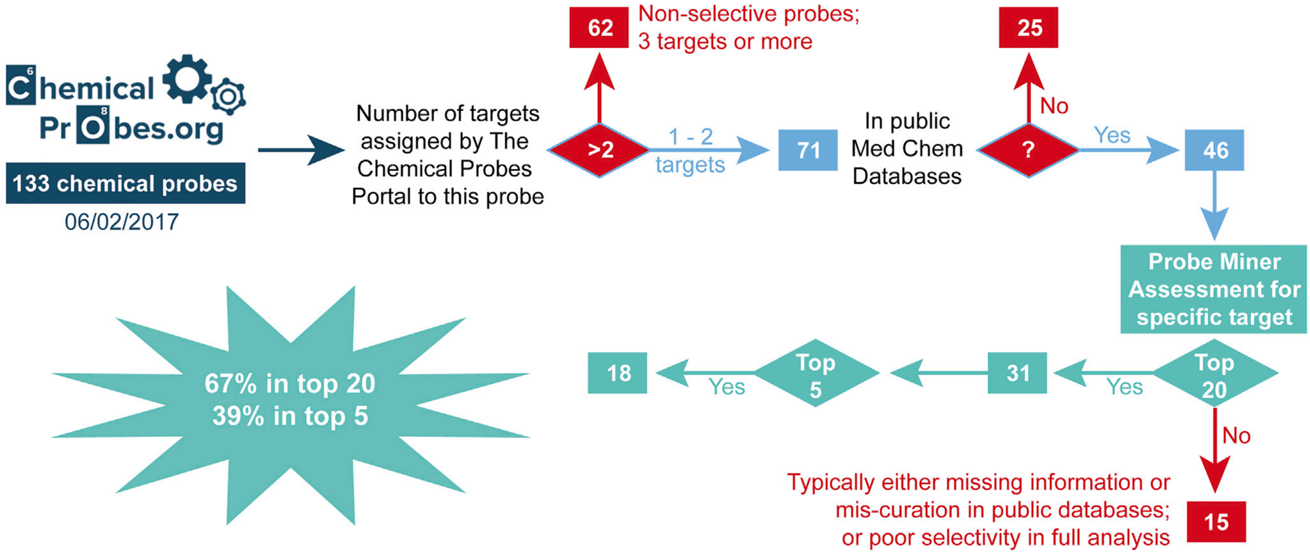


**Figure 4. Flow Diagram for the Comparison between Probe Miner and the Chemical Probes Portal**

## Use Cases: PARP1, CHEK2, OPRK1, and ABCC8

We have selected four use cases to illustrate the value and also current limitations of the Probe Miner resource.

### *PARP1*

The cancer drug target poly(ADP-ribose) polymerase 1 (PARP1) DNA repair enzyme has five pan-PARP probes that are recommended by the Chemical Probes Portal. Olaparib, veliparib, and niraparib are all highly ranked using Probe Miner's predefined Global Score (Figures 3 and 5). However, key information is missing in public resources regarding the Portal-listed probes AZ0108 and E7449, the latter published in a journal not indexed in public databases (McGonigle et al., 2015). Accordingly, these two probes are not highly ranked in Probe Miner (Figure 5). On the other hand, our objective assessment resource identifies another probe that scores highly but has not yet been curated by the Chemical Probes Portal. This is NMS-P118, a recently published PARP1-selective inhibitor that was comprehensively screened for kinase selectivity (Papeo et al., 2015), which is very important given reports of off-target activity against kinases among PARP inhibitors (Antolín and Mestres, 2014). Therefore, NMS-P118 emerges as a potential candidate with which to probe specifically for PARP1 (Figure 5). Based on our findings, we proposed NMS-P118 to the Chemical Probes Portal, and this chemical probe is now under review for expert curation.

### *CHEK2*

The serine/threonine-protein kinase CHEK2, a cell-cycle checkpoint protein, exemplifies errors and limitations in the public medicinal chemistry data resources. For CHEK2, use of our Global Score initially failed to prioritize the selective chemical probe CCT241533 (Caldwell et al., 2011), which is expert-curated in the Chemical Probes Portal, while ranking as the highest-scoring chemical tool the very broadly characterized but promiscuous kinase inhibitor, sunitinib. We found that CCT241533 had fallen foul of a series of errors and missing data in public medicinal chemistry databases. Most significantly, the affinity of CCT241533 for CHEK2 had been wrongly curated in ChEMBL, making the probe appear non-selective (Figure S4). We reported this error to ChEMBL and it has now been corrected in both ChEMBL and our canSAR database. As a result, CCT241533 now ranks as the third highest-scoring probe in Probe Miner (Figure 5).

In considering examples like this, it is important to note that our Selectivity Score balances the contribution of actually measured selectivity and also the extent of characterization for potential off-targets. For example, if one probe appears selective but has only been tested against two off-targets, while another probe is not completely selective but has been tested against hundreds of targets, then our Selectivity Score may reward the more widely characterized probe (see STAR Methods for details). This is the case in our analysis of sunitinib as a probe. The very thorough characterization of sunitinib against the kinome is the key factor that results in it ranking as the top probe when using our predefined weightings for the Global Score (see STAR Methods and Figure S5). This further emphasizes the importance of carefully evaluating all data available, and, importantly, of expert curation where possible, before selecting any chemical probe, regardless of its ranking in Probe Miner.

Furthermore, selectivity information is inconsistently reported in public databases, making the data difficult to automatically extract and compare (e.g., selectivity is sometimes reported as % inhibition or activity at different concentrations, or even °C from differential scanning fluorimetry measurements, rather than bioactivity or binding affinity measured in molar concentrations). Our Probe Miner algorithm currently uses only selectivity data reported in molar concentrations. Although interpretable to a human expert, there is no globally applicable computational method to convert % inhibition data to comparable half maximal inhibitory concentration ($IC_{50}$) at scale. Consequently, where selectivity data are captured as % inhibition, as for CCT241533, which was tested against a panel of 85 kinases, these data are not incorporated into our current Selectivity Score.

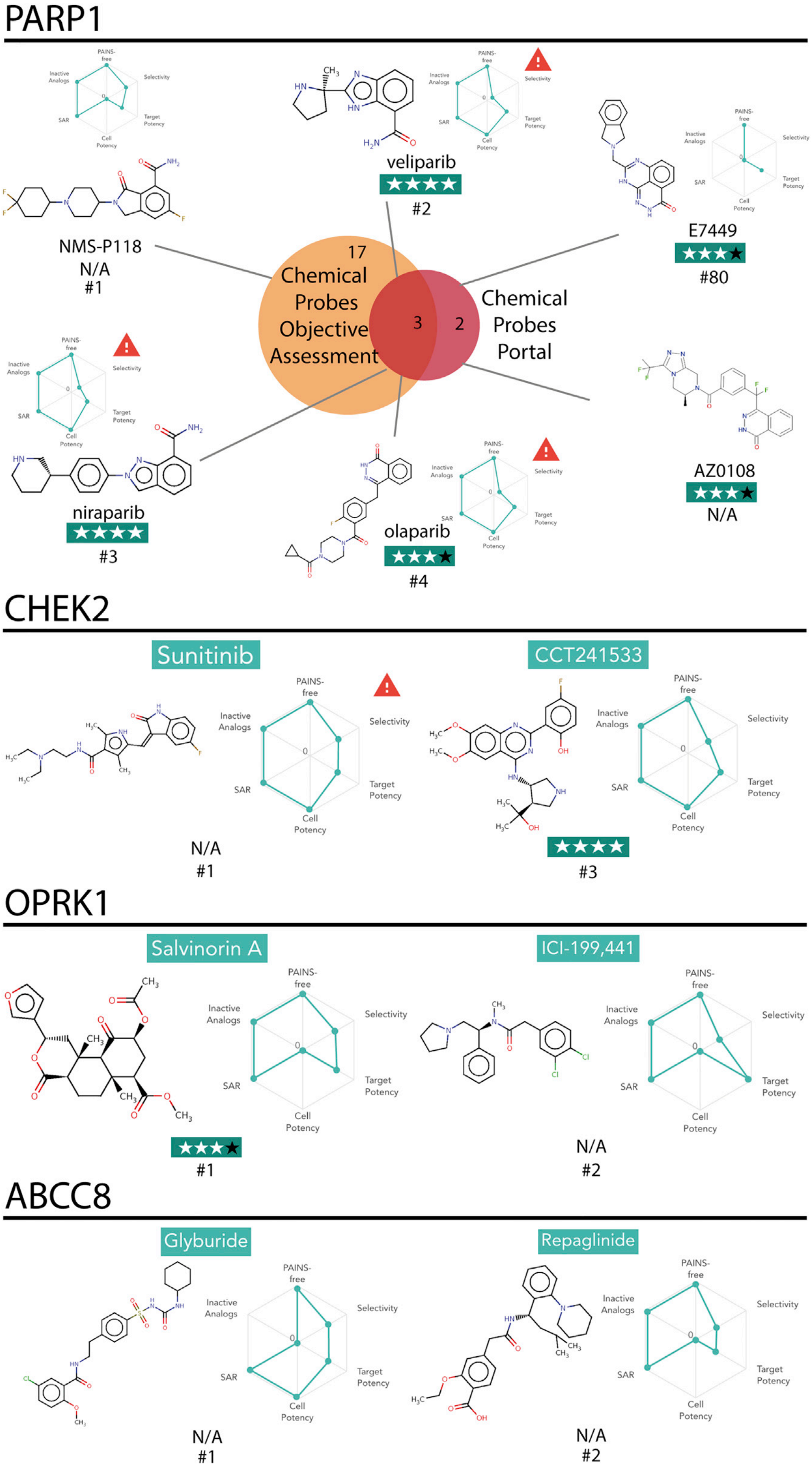


**Figure 5. Analysis of the Ranking of Chemical Probes for the Targets PARP1, CHEK2, and OPRK1**

On top, Venn diagram comparing the PARP1 chemical probes recommended by the Chemical Probes Portal (see STAR Methods) and the Probe Miner resource as ranked by the predefined Global Score. Chemical structures are displayed, as well as names, a radar plot showing the six Chemical Probe scores, the Chemical Probes Portal reviewers' rating, and Probe Miner ranking when available. On the bottom, highest-ranked probes for CHEK2, OPRK1 and ABCC8. See also Figure S4.

### *OPRK1*

The use of more than one chemical probe with different chemotypes is strongly recommended for mechanistic studies and target validation (Blagg and Workman, 2017). Accordingly, our third example, the Kappa opioid receptor OPRK1, illustrates how Probe Miner can be used to identify second probes and potentially contribute to increase the completeness of the Chemical Probes Portal.

For OPRK1, there is only one chemical probe currently covered in the Portal, namely the natural product agonist salvinorin A. While Probe Miner does identify salvinorin A as the highest-ranking probe for OPRK1, it also identifies chemically distinct probes for this target, such as the drug ICI-199,441 (Weerawarna et al., 1994), which is a potent, selective, and commercially available agonist and can thus be used in conjunction with salvinorin A to probe the biology of the OPRK1 receptor (Figure 5). Thus, we have recommended the OPRK1 chemical probe ICI-199,441 for consideration by the Chemical Probes Portal for expert review that would complement our large-scale, data-driven objective assessment in Probe Miner while increasing the coverage of OPRK1 inhibitors by the Portal.

### *ABCC8*

ABCC8 (ATP-binding cassette sub-family C member 8) functions as a modulator of ATP-sensitive potassium channels and insulin release and serves as an example of how Probe Miner's broader coverage across the whole liganded proteome can be used to aid in the prioritization and selection for expert curation of probes for targets that are currently not yet included in the Chemical Probes Portal. Our objective assessment of ABCC8, a subunit of the beta cell ATP-sensitive potassium channel (KATP), identifies the inhibitory, antidiabetic drugs glyburide and repaglinide as the highest-ranking probes (Coghlan et al., 2001). These two agents are commercially available and could thus be readily used to probe for this ion channel while waiting for more in-depth expert curation of probes for this target at the Portal.

Taken together, these four use cases discussed above represent typical scenarios that highlight the synergy and complementarity of the Probe Miner and Chemical Probes Portal resources in chemical probe selection.

## DISCUSSION

Chemical probes are an essential part of functional genome annotation, mechanistic exploration of biology and disease, and validation of drug targets, but there are serious issues with their quality, selection, and use (Blagg and Workman, 2017). Here, we report: (1) our data-driven, unbiased, objective analysis of large-scale public data to catalog currently available tools and evaluate potential chemical probes; (2) our exemplification of the utilization and value of the approach as well as the limitations imposed by the current nature of the underlying data; and (3) our description of the use of Probe Miner, which we have made freely available to the research community as a public resource to facilitate the identification and prioritization of potential chemical probes that should be evaluated further.

Through our systematic analysis of >3.9 million experimental activities for >1.8 million compounds in curated public medicinal chemistry databases, we provide objective, data-driven systematic scoring of 355,305 compounds against 2,220 human protein targets. Using our data-driven assessment, we provide quantitative data demonstrating the extent to which the majority of human proteins lack minimal quality small-molecule chemical tools that are needed to probe their function. In addition, we demonstrate gaps in the description and characterization of chemical tools in public databases, we highlight the severity of our limited knowledge of chemical tool selectivity and we uncover large historical biases in the reported exploration of chemical space and the polypharmacology of active compounds, resulting in disparity in the number of minimal quality probes identifiable for different targets. It is therefore paramount that the chemical biology community improves the quality and especially the degree of broad characterization of currently available chemical tools across multiple targets, and in addition also continues the discovery and characterization of novel tools for as yet non-probed proteins.

Thus, while recognizing that the number, quality, and proteome-wide coverage of chemical probes will increase with time, especially the important factor of degree of selectivity profiling, Probe Miner provides an additional and distinct resource that will be useful both today and in the future to help empower researchers to select the best tools available for biomedical research. We believe that our systematic, objective assessment resource, derived from the underlying, evolving large-scale medicinal chemistry data, is an important addition to the toolbox for chemical probe prioritization. Probe Miner can be used by the community to fill the gap while expert-curation approaches such as the Chemical Probes Portal expand into protein families that have not yet been covered. It can also be employed to help prioritize probes for subsequent expert curation and assessment at the Portal or by individual researchers. Moreover, we show that our unbiased large-scale approach, which benefits from regular, automatic updates, will be especially powerful when combined with the complementary expert-curated assessments provided by the Chemical Probes Portal (Arrowsmith et al., 2015; www.chemicalprobes.org). When used together with the experience, knowledge, and opinion of the individual investigator, Probe Miner and the Portal provide both the breadth and depth required to make informed choices on the selection of chemical probes. In practice, individual researchers may in some cases have to make the choice between a well-characterized but not optimally selective probe versus a less well-characterized but seemingly selective one. As illustrated by the examples shown, the combined resources help ensure that investigators are fully aware of available knowledge and gaps therein. Note also that, in any case, expert guidelines recommend the combined use of at least two chemical probes from distinct chemotypes, together with at least one inactive control compound (Blagg and Workman, 2014; Workman and Collins, 2010). Overall, Probe Miner and the Chemical Probes Portal have complementary strengths which will make their continued combined use synergistic and mutually beneficial to the user community.

Probe Miner represents, to our knowledge, the first publicly available resource enabling objective, data-driven, systematic assessment of chemical tools. We demonstrate that our data-led prioritization of chemical tools aligns well with expert recommendation from the Chemical Probes Portal when both approaches have access to the same information. However,

we also uncover incompleteness, inaccuracies, and inconsistencies of data deposited in public databases that limit the full benefit of our large-scale objective approach. An important point to note is that the public databases used in this analysis were developed mainly for medicinal chemistry applications and, accordingly, many chemical biology publications are not covered. Moreover, we have identified errors in public databases that have now been corrected (Figure S4). We also found several inaccuracies, particularly regarding the annotation of cell-based half maximal effective concentration ($EC_{50}$) values and biochemical $IC_{50}$ values, as well as inconsistencies regarding the deposition of selectivity data in public databases (Table S3). Therefore, there is a great need to better capture and curate medicinal chemistry and chemical biology information from the literature in public knowledge bases if we are to make the most of these expensively generated data. Such improvements will also allow further evolution of Probe Miner algorithm over time.

We stress that our Probe Miner resource can be used either with a predefined weighting of fitness factors, or with user-customised weights. By default, measures of biochemical activity/binding potency and selectivity, as well as surrogate measures of cell permeability, are given greater weighting in the overall Global Score. The preset default weightings for assessing and ranking chemical probes may be especially useful for biomedical scientists who are not chemical biology or medicinal chemistry experts, which represents a large and important community that was highlighted recently as requiring advice and user-friendly resources when selecting chemical tools for exploring biology and conducting target validation (Blagg and Workman, 2017). Alternatively, the weighting of different criteria can be customized according to individual researchers' views and needs. For example, expert users may wish to alter the weighting of fitness factors to suit their own experience and opinion. Or they may wish to vary the weightings of different factors to see how this affects the ranking of probes.

In conclusion, we demonstrate here that objective, quantitative, data-driven large-scale assessment based on public data can contribute to improving overall evaluation and prioritization of chemical probes. We propose that our new Probe Miner resource represents a valuable contribution for the identification of potential chemical probes, particularly when used alongside expert curation.

## SIGNIFICANCE

**The selection of appropriate chemical probes is essential for mechanistic biological investigation and target validation but continues to be largely biased and subjective and does not benefit from the large-scale data available in public databases. Here, we statistically analyze >1.8 million compounds and >2,200 human targets. Our objective study provides insights that can be used to assess and select chemical probes. It also uncovers significant biases in the exploration of chemical probes in public databases. To enable an objective and quantitative assessment of chemical probes, we have developed data-driven probe scoring metrics aligned to key properties or fitness factors. To empower the community to utilize this knowledge, we have also developed the Probe Miner resource (http://probeminer.icr.ac.uk) to enable public access to this information and algorithm in a user-friendly framework. We demonstrate how our objective assessment generally aligns with expert recommendation from the Chemical Probes Portal when the information in public databases is available and accurate, and also provides synergistic benefits, for example, through its scale, objectivity, and lack of bias, and also its quantitative nature. Importantly, we provide examples showing how our data-driven assessment can inform selection of probes for expert curation. Thus, Probe Miner represents a valuable resource to empower the chemical biology and general research community toward the selection of high-quality chemical probes for mechanistic studies and target validation.**

## STAR★METHODS

Detailed methods are provided in the online version of this paper and include the following:

- KEY RESOURCES TABLE
- CONTACT FOR REAGENT AND RESOURCE SHARING
- METHOD DETAILS
  - Definitions
  - Chemical Probe Scores and Commercial Availability
  - Development of the Probe Miner: Chemical Probes Objective Assessment Resource
  - Analysis of the 'Liganded' Proteome and Chemical Tools for Cancer Genes
  - Analysis of Chemical Probes from The Chemical Probes Portal
- DATA AND SOFTWARE AVAILABILITY
- ADDITIONAL RESOURCES

## SUPPLEMENTAL INFORMATION

Supplemental Information includes six figures and three tables and can be found with this article online at https://doi.org/10.1016/j.chembiol.2017.11.004.

## AUTHOR CONTRIBUTIONS

A.A.A., I.C., P.W., and B.A.-L. designed the research. A.A.A. performed the experimental work. A.A.A., I.C., P.W., and B.A.-L. conducted data analysis and interpretation. A.A.A., J.E.T., A.K., and B.A.-L. designed the website. J.E.T and A.K. coded and developed the website resource. A.A.A., I.C., P.W., and B.A.-L. contributed to manuscript preparation.

## ACKNOWLEDGMENTS

This work was primarily supported through the People Program (Marie Curie Actions) of the 7th Framework Program of the European Union (FP7/2007-2013) under REA grant agreement no. 600388 (TECNIOspring program); the Agency of Business Competitiveness of the Government of Catalonia, ACCIÓ to A.A.A.; and the Wellcome Trust Sir Henry Wellcome Postdoctoral Fellowship (204735/Z/16/Z) to A.A.A. The work utilizes the canSAR knowledge base, which is funded by a Cancer Research UK (CRUK) Strategic Project Award (C35696/A23187) to B.A.-L. In addition, B.A.-L., I.C., and P.W. are funded by The Institute of Cancer Research (ICR) as well as the Cancer Research CRUK program grant to the Cancer Research UK Cancer Therapeutics Unit (grant C309/A11566). P.W is a CRUK Life Fellow. We acknowledge support from CRUK and NHS funding to the CRUK Centre and Biomedical Research Centre, respectively, at the ICR and Royal Marsden NHS Foundation Trust. The authors thank many collaborators for discussions and valuable input into the preparation of

this manuscript. In particular, the authors thank Amy Donner for helpful discussion with regard to the Chemical Probes Portal and Gary Nugent for facilitating the chemoinformatics scripts to calculate the PAINS and the Level 1 scaffolds. We also thank early Probe Miner users for valuable feedback. A.A.A., J.E.T., A.K., I.C., P.W., and B.A.-L. are employees of The Institute of Cancer Research (ICR), which has a commercial interest in a range of drug targets. The ICR operates a Rewards to Inventors scheme through which employees of the ICR may receive financial benefit following commercial licensing of a project. P.W and I.C. are members of the Scientific Advisory Board of the non-profit Chemical Probes Portal and P.W. is also a Board Director.

Received: August 8, 2017
Revised: September 22, 2017
Accepted: November 14, 2017
Published: December 14, 2017

## REFERENCES

Andrs, M., Korabecny, J., Jun, D., Hodny, Z., Bartek, J., and Kuca, K. (2015). Phosphatidylinositol 3-kinase (PI3K) and phosphatidylinositol 3-kinase-related kinase (PIKK) inhibitors: importance of the morpholine ring. J. Med. Chem. *58*, 41–71.

Antolín, A.A., and Mestres, J. (2014). Linking off-target kinase pharmacology to the differential cellular effects observed among PARP inhibitors. Oncotarget *5*, 1–6.

Arrowsmith, C.H., Audia, J.E., Austin, C., Baell, J., Bennett, J., Blagg, J., Bountra, C., Brennan, P.E., Brown, P.J., Bunnage, M.E., et al. (2015). The promise and peril of chemical probes. Nat. Chem. Biol. *11*, 536–541.

Baell, J., and Walters, M.A. (2014). Chemical con artists foil drug discovery. Nature *513*, 481–483.

Blagg, J., and Workman, P. (2014). Chemical biology approaches to target validation in cancer. Curr. Opin. Pharmacol. *17*, 87–100.

Blagg, J., and Workman, P. (2017). Choose and use your chemical probe wisely to explore cancer biology. Cancer Cell *32*, 9–25.

Bulusu, K.C., Tym, J.E., Coker, E.A., Schierz, A.C., and Al-Lazikani, B. (2014). CanSAR: updated cancer research and drug discovery knowledgebase. Nucleic Acids Res. *42*, 1040–1047.

Bunnage, M.E., Chekler, E.L.P., and Jones, L.H. (2013). Target validation using chemical probes. Nat. Chem. Biol. *9*, 195–199.

Caldwell, J.J., Welsh, E.J., Matijssen, C., Anderson, V.E., Antoni, L., Boxall, K., Urban, F., Hayes, A., Raynaud, F.I., Rigoreau, L.J.M., et al. (2011). Structure-based design of potent and selective 2-(quinazolin-2-yl)phenol inhibitors of checkpoint kinase 2. J. Med. Chem. *54*, 580–590.

Capuzzi, S.J., Muratov, E.N., and Tropsha, A. (2017). Phantom PAINS: problems with the utility of alerts for Pan-Assay INterference CompoundS. J. Chem. Inf. Model. *57*, 417–427.

Chambers, J., Davies, M., Gaulton, A., Hersey, A., Velankar, S., Petryszak, R., Hastings, J., Bellis, L., McGlinchey, S., and Overington, J.P. (2013). UniChem: a unified chemical structure cross-referencing and identifier tracking system. J. Cheminform. *5*, 1–9.

Coghlan, M.J., Carroll, W.A., and Gopalakrishnan, M. (2001). Recent developments in the biology and medicinal chemistry of potassium channel modulators: update from a decade of progress. J. Med. Chem. *44*, 1627–1653.

Edwards, A.M., Isserlin, R., Bader, G.D., Frye, S.V., Willson, T.M., and Yu, F.H. (2011). Too many roads not taken. Nature *470*, 163–165.

Fedorov, O., Müller, S., and Knapp, S. (2010). The (un)targeted cancer kinome. Nat. Chem. Biol. *6*, 166–169.

Finan, A.C., Gaulton, A., Kruger, F.A., Lumbers, T., Shah, T., Engmann, J., Galver, L., Kelley, R., Karlsson, A., Santos, R., et al. (2017). The druggable genome and support for target identification and validation in drug development. Sci. Transl. Med. *9*, 383.

Folkes, A.J., Ahmadi, K., Alderton, W.K., Alix, S., Baker, S.J., Box, G., Chuckowree, I.S., Clarke, P.A., Depledge, P., Eccles, S.A., et al. (2008). The identification of 2-(1H-indazol-4-yl)-6-(4-methanesulfonyl-piperazin-1-ylmethyl)-4-morpholin-4-yl-thieno[3,2-d]pyrimidine (GDC-0941) as a potent, selective, orally bioavailable inhibitor of class I PI3 kinase for the treatment of cancer. J. Med. Chem. *51*, 5522–5532.

Frye, S.V. (2010). The art of the chemical probe. Nat. Chem. Biol. *6*, 159–161.

Gaulton, A., Hersey, A., Nowotka, M., Bento, A.P., Chambers, J., Mendez, D., Mutowo, P., Atkinson, F., Bellis, L.J., Cibrián-Uhalte, E., et al. (2016). The ChEMBL database in 2017. Nucleic Acids Res. *45*, gkw1074.

Gilson, M.K., Liu, T., Baitaluk, M., Nicola, G., Hwang, L., and Chong, J. (2016). BindingDB in 2015: a public database for medicinal chemistry, computational chemistry and systems pharmacology. Nucleic Acids Res. *44*, D1045–D1053.

Hopkins, A.L., Keserü, G.M., Leeson, P.D., Rees, D.C., and Reynolds, C.H. (2014). The role of ligand efficiency metrics in drug discovery. Nat. Rev. Drug Discov. *13*, 105–121.

Huggins, D.J., Venkitaraman, A.R., and Spring, D.R. (2011). Rational methods for the selection of diverse screening compounds. ACS Chem. Biol. *6*, 208–217.

Langdon, S.R., Brown, N., and Blagg, J. (2011). Scaffold diversity of exemplified medicinal chemistry space. J. Chem. Inf. Model. *51*, 2174–2185.

Mateo, J., Ganji, G., Lemech, C., Burris, H.A., Han, S.-W., Swales, K.E., Decordova, S., DeYoung, M.P., Smith, D.A., Kalyana-Sundaram, S., et al. (2017). A first-time-in-human study of GSK2636771, a phosphoinositide 3 kinase beta-selective inhibitor, in patients with advanced solid tumors. Clin. Cancer Res. *23*, 5981–5992.

McGonigle, S., Chen, Z., Wu, J., Chang, P., Kolber-Simonds, D., Ackermann, K., Twine, N.C., Shie, J.-L., Miu, J.T., Huang, K.-C., et al. (2015). E7449: a dual inhibitor of PARP1/2 and tankyrase1/2 inhibits growth of DNA repair deficient tumors and antagonizes Wnt signaling. Oncotarget *6*, 41307–41323.

Oprea, T.I., Bologa, C.G., Boyer, S., Curpan, R.F., Glen, R.C., Hopkins, A.L., Lipinski, C.A., Marshall, G.R., Martin, Y.C., Ostopovici-Halip, L., et al. (2009). A crowdsourcing evaluation of the NIH chemical probes. Nat. Chem. Biol. *5*, 441–447.

Papeo, G., Posteri, H., Borghi, D., Busel, A.A., Caprera, F., Casale, E., Ciomei, M., Cirla, A., Corti, E., D'Anello, M., et al. (2015). Discovery of 2-[1-(4,4-difluorocyclohexyl)piperidin-4-yl]-6-fluoro-3-oxo-2,3-dihydro-1H-isoindole-4-carboxamide (NMS-P118): a potent, orally available, and highly selective PARP-1 inhibitor for cancer therapy. J. Med. Chem. *58*, 6875–6898.

Raynaud, F.I., Eccles, S., Clarke, P.A., Hayes, A., Nutley, B., Alix, S., Henley, A., Di-Stefano, F., Ahmad, Z., Guillard, S., et al. (2007). Pharmacologic characterization of a potent inhibitor of class I phosphatidylinositide 3-kinases. Cancer Res. *67*, 5840–5850.

Rubio-Perez, C., Tamborero, D., Schroeder, M.P., Antolín, A.A., Deu-Pons, J., Perez-Llamas, C., Mestres, J., Gonzalez-Perez, A., and Lopez-Bigas, N. (2015). In silico prescription of anticancer drugs to cohorts of 28 tumor types reveals targeting opportunities. Cancer Cell *27*, 382–396.

Sanchez, R.M., Erhard, K., Hardwicke, M.A., Lin, H., McSurdy-Freed, J., Plant, R., Raha, K., Rominger, C.M., Schaber, M.D., Spengler, M.D., et al. (2012). Synthesis and structure–activity relationships of 1,2,4-triazolo[1,5-a]pyrimidin-7(3H)-ones as novel series of potent β isoform selective phosphatidylinositol 3-kinase inhibitors. Bioorg. Med. Chem. Lett. *22*, 3198–3202.

Schreiber, S.L. (2005). Small molecules: the missing link in the central dogma. Nat. Chem. Biol. *1*, 64–66.

The UniProt Consortium (2017). UniProt: the universal protein knowledgebase. Nucleic Acids Res. *45*, 158–169.

Tym, J.E., Mitsopoulos, C., Coker, E.A., Parisa, R., Schierz, A.C., Antolin, A.A., and Al-Lazikani, B. (2016). CanSAR: an updated cancer research and drug discovery knowledgebase. Nucleic Acids Res. *44*, 938–943.

Wang, Y., Cornett, A., King, F.J., Mao, Y., Nigsch, F., Paris, C.G., McAllister, G., and Jenkins, J.L. (2016). Evidence-based and quantitative prioritization of tool compounds in phenotypic drug discovery. Cell Chem. Biol. *23*, 862–874.

Weerawarna, S.A., Davis, R.D., and Nelson, W.L. (1994). Isothiocyanate-substituted/c-selective opioid receptor ligands derived from N-Me thyl-N-[(1s)-1-phenyl-2-(1-pyrrolidinyl)ethyl] phenylace tamide. J. Med. Chem. *13*, 2856–2864.

Workman, P., and Collins, I. (2010). Probing the probes: fitness factors for small molecule tools. Chem. Biol. *17*, 561–577.

# STAR★METHODS

## KEY RESOURCES TABLE

| REAGENT or RESOURCE | SOURCE | IDENTIFIER |
|---|---|---|
| Deposited Data | | |
| ChEMBL22 | Gaulton et al., 2016 | https://www.ebi.ac.uk/chembl/; RRID: SCR_014042 |
| BindingDB | Gilson et al., 2016 | https://www.bindingdb.org; RRID: SCR_000390 |
| canSAR v3 | Tym et al., 2016 | http://cansar.icr.ac.uk/; RRID: SCR_006794 |
| Software and Algorithms | | |
| Scaffold Tree Algorithm (implemented using Pipeline Pilot, Dassault Systèmes BIOVIA, Pipeline Pilot, 9.5, San Diego: Dassault Systèmes, 2017) | Langdon et al., 2011 | http://probeminer.icr.ac.uk/#/download |
| PAINS FILTERS (Implemented using VORTEX), Adapted from Chris Swain's MacInChemBlog | Baell and Walters, 2014 | https://www.macinchem.org/reviews/pains/painsFilter.php |

## CONTACT FOR REAGENT AND RESOURCE SHARING

Further information and requests for resources should be directed to and will be fulfilled by the Lead Contact, Bissan Al-Lazikani (Bissan.Al-Lazikani@icr.ac.uk).

## METHOD DETAILS

### Definitions

During this work we have used the following definitions:

#### *Target*

Human protein that is known to interact with a chemical compound.

#### *Reference Target*

Since a chemical compound can bind to multiple protein targets and we score each compound-target pair, the reference target is defined as the target of the compound that is being evaluated.

#### *Potency Score*

Score that measures the potency of the biochemical interaction between each compound-target pair.

#### *Selectivity Score*

Score that measures the selectivity of each compound-target pair. Selectivity is one of the most important properties that a chemical tool should fulfil in order to be useful to study the biological function and therapeutic potential of a specific protein (Frye, 2010; Workman and Collins, 2010). However, it is challenging to measure due to large biases in the number of targets screened for each compound (Figure S5A) and thus the selectivity score balances our actual knowledge of selectivity with the amount of selectivity information that is actually available.

#### *Cell Score*

Since no large-scale experimental measure of permeability is available, we use cellular activity as a proxy. Accordingly, the Cell Score is a binary score that measures whether a given chemical molecule is known to be active in cells, and thus accounts not only for the permeability but also for the solubility and cell activity fitness factors because when a compound is active in a cell line assay we assume it fulfils minimum requirements of permeability and solubility to modulate the target of interest in cells (Workman and Collins, 2010).

#### *SAR Score*

Structure-Activity Relationships (SAR) increase confidence that the biological effect of a given chemical tool is achieved via the modulation of the reference target. Accordingly, the SAR score is a binary score measuring whether there are (SAR Score = 1) known SAR for the compound-reference target pair (Workman and Collins, 2010).

#### *Inactive Analog Score*

Inactive analogs can be useful controls to rule out off-target effects. Therefore, the Inactive Analog Score is a binary score measuring whether there are known inactive analogs for the compound-reference target pair (Workman and Collins, 2010).

#### *PAINS Score*

Pan-assay interference compounds (PAINS) are those that interfere with the detection methods of screening assays and are thus problematic artefacts that have been identified to be widely used in many scientific publications as chemical tools, thus leading to the wrong conclusions (Baell and Walters, 2014). There are several computational methods that can be used to predict PAINS

and other potentially problematic functional groups in biomedical and cell-based chemical tools (Huggins et al., 2011). However, none of the available computational methods is exempt of limitations, among them a considerable number of false-positives (Capuzzi et al., 2017; Huggins et al., 2011). Accordingly, the PAINS Score measures whether a compound is predicted to be PAINS-free using the substructure filters proposed by Baell et al. (see STAR Methods) as a pragmatic means to alert users to the potential risk of functionality in the molecule that may cause misleading effects in biochemical or cell-based assays. However, to reduce the impact of false-positives we give this PAINS Score a low weighting in the default Global Score to avoid deprioritising otherwise promising molecules. Still, it is worth highlighting that the PAINS Score is a prediction calculated using only one computational method. Thus, we enable researchers to set the weight of the PAINS Score in the Global Score according to their expert judgement and the specific assay that they are using. Moreover, researchers can also remove the PAINS Score from the calculation of the Global Score by setting the weight to 0.

## Chemical Probe Scores and Commercial Availability

### *Potency Score*

We consider bioactivity data on compound-target pairs integrated in the knowledgebase canSAR (that integrates high quality bioactivity data from CHEMBL and BindingDB) (Gaulton et al., 2016; Gilson et al., 2016) where the target type is a protein, the protein is human as defined by its associated Uniprot ID and the units can be transformed to ‘nM’ (The Uniprot Consortium, 2017; Tym et al., 2016). We calculate the median of all the reported values for each compound-target pair distinguishing between ‘=’ and ‘>’ values and transform them into pActivity values (-log(Activity[Molar])). We consider active all compounds with a median pActivity below 10,000 nM. Compounds with conflicting ‘=’ and ‘>’ data for the same target are considered inactive. Given that only 0.7% of bioactivities are below 100 picomolar but they strongly bias the normalisation of the potency score, these activity values (pActivity > 10) are given a value of 10. For all the compound-target pairs considered as active, the potency score is calculated as the normalization of the pActivity values in a scale from 0 (pActivity = 5) to 1 (pActivity $\geq$ 10). There have been arguments against any numerical aggregation of potency values due to their wide variation across biological systems and technologies, but the proposed alternative uses subjective expert weighting of several databases including highly curated proprietary databases that are not widely accessible (Wang et al., 2016). We have thoroughly investigated cases where a wide distribution of potency values has been reported, such as nilotinib (CHEMBL255863; Wang et al., 2016). We identify that these wide distributions are often due to inaccuracies in the annotation of cellular $EC_{50}$ values as biochemical $IC_{50}$ values and the annotation of data from mutants as wild type proteins (Gaulton et al., 2016). We have calculated the Median Absolute Deviation (MAD) of the potency median calculated for each compound-target pair and we identify that these large variations affect only a very small number of compounds (3.6%; Figure S6). We provide the MAD to facilitate the identification of cases where this wide distribution may affect the reported performance of the chemical tool. These results support the use of high quality public databases for the assessment of chemical tools and highlight the need to better curate these high quality databases to make the most of this expensively generated data.

### *Selectivity Score*

For each compound, we calculate a different selectivity score for each of the proteins it interacts with (median pActivity > 5) considering all of their compound-target interactions. In order to balance the actual knowledge of selectivity with the amount of information available (Figure S5A), the Selectivity Score is composed by three different factors in an attempt to reflect our limited cataloguing of selectivity following the formula below:

$$Selectivity\ Score = \frac{First\ Factor + Second\ Factor_{normaized\ per\ target} + Third\ Factor}{3}$$

The First Factor accounts for the actual knowledge of selectivity. In order to calculate the First Factor, the number of off-targets that the compound has been screened against is calculated first, without including the target being evaluated. Second, the median pActivity values are used to discern whether there is at least 10-fold selectivity (1 log unit) between the potency of the compound for the reference target and the potency for each off-target ($pActivity_{ReferenceTarget} - pActivity_{Off\text{-}Target} \geq 1$). The 10-fold selectivity cut-off has been previously used as the minimum selectivity requirement to consider that a chemical probe is selective (Oprea et al., 2009). If there is 10-fold selectivity between the potency of the compound for the reference target and the potency of the off-target, this off-target is considered a selective off-target (Figure S5B). Next, we calculate the number of selective off-targets. The First Factor of the Selectivity Score is obtained by dividing the number of selective off-targets by the total number of off-targets, using the following equation:

$$First\ Factor = \frac{Number\ of\ 10\_fold\ selective\ off\_targets}{Total\ number\ of\ off\_targets}$$

Therefore, if a compound-reference target interaction is at least 10-fold selective against any other target, the value of the first factor will be 1. In contrast, if the compound-target interaction of interest is not 10-fold selective against any other compound-target interactions, the first factor will be lower than 1 (Figure S5B). If there is no information regarding any off-target, the Selectivity Score is set to 0.

The second factor of the score is a measure of the amount information available regarding selectivity. This second factor distinguishes between compounds that have an equal first factor but have been screened against a (very) different number of targets. Moreover, it also balances the actual knowledge of selectivity – measured by the first factor – with the amount of information available

regarding selectivity that can be very different between different compounds, challenging their comparison (Figures S5B and S5C). In order to calculate it, we have developed a measure of selectivity information that we have termed Selectivity Information Richness (SIC). The SIC is calculated as the summary of the differences between the median pActivity of the reference target and the pActivity of each off-target minus one:

$$SIC = \sum_{i=1}^{number\ of\ off-targets} \left(pActivity_{Target\ of\ Interest} - pActivity_{off\_Target\ i} - 1\right)$$

The above approach enables the evaluation of the selectivity information from the 10-fold selectivity cut-off in order to distinguish selective from non-selective information that will be positive and negative, respectively (Figure S5C). Therefore, in the final summary unselective data compensate for selective data. Interestingly, the SIC could also be regarded as the number of selectivity units from the given compound-protein interaction. To calculate the Second Factor of the Selectivity Score, the SIC is divided by the number of targets that would be modulated at the same time since there is not 10-fold selectivity between them, therefore the number of not 10-fold selective off-targets plus the target of interest, following this formula:

$$Second\ Factor = \frac{SIC}{Number\ of\ not\ 10 - fold\ selective\ off\_targets + 1}$$

This division enables reduction the SIC of compound-target interactions that are very high, as the compound has been screened against a very large number of off-targets, but represent suboptimal compounds to probe for the target of interest as they inhibit several other targets with similar or higher affinity (Figure S5D).

The second factor is finally normalized within each target as we observe that different targets can have very different SIC ranges. The main reason for this is that there are target families such as kinases where family-wide profiling is very common while this is not common for other target families and a global normalization would profoundly bias the results.

Finally, the last factor measures the percentage of the proteome that the compound has been screened against, which is generally very low, with the following formula:

$$Third\ Factor = \frac{Number\ of\ off\_targets + 1}{Number\ of\ liganded\ targets}$$

Therefore, this Third Factor serves as a reminder that the selectivity of chemical tools is generally a major unknown. Ultimately, the three factors are added and the final score is normalized (Equation 1). Overall, the Selectivity Score is able to balance different aspects of selectivity; however, how compounds screened against a very different number of targets should be prioritized remains a difficult question. Our aim in developing the selectivity score was to prioritize compounds and facilitate the evaluation of the information available but the final decision should always be taken by the researcher after careful evaluation of available information and tailored to the requirements of the specific question.

#### Cell Score

To calculate the Cell Score we compute the median of all compound- cell line bioactivities reported in canSAR that can be transformed to ‘nM’ (Tym et al., 2016). We consider a compound has positive Cell Score (Score =1) if it is active in at least one cell line considering a cut-off of 10,000 nM (median pActivity > 5). This cut-off is set to minimise the risk of considering non-specific drug toxicity that may lead to cell death at high concentrations. Compounds that have activity values less potent that the cut-off or that have not been tested in cell line assays are given a Cell Score value of 0.

#### SAR Score

To calculate the SAR Score we first calculate the level 1 of the scaffold tree for all compounds in canSAR as it has been described to have advantages over other scaffold definitions (Langdon et al., 2011). Next, we consider a compound-reference target pair has SAR (SAR Score = 1) if there is at least another compound reported in the same publication (identical PubMedID) with the same level 1 scaffold active against the reference target (pActivity > 5).

#### Inactive Analog Score

The Inactive Analog Score measures whether there are compounds sharing the level 1 scaffold with the compound being evaluated that are reported to be inactive (pActivity < 5) for the reference target.

#### PAINS Score

We apply PAINS rules to filter compounds that are given a PAINS alert by giving them a PAINS score of 0 (Baell and Walters, 2014).

#### Global Chemical Probe Score

The Global Chemical Probe Score is a combination of the previous 6 Chemical Probe Scores with customizable weights to allow chemical biologists to prioritize the best chemical tools for the specific requirements of their experiments. We have predefined weights for a case where selectivity is twice as important as potency, which in turn is twice as important as cell activity, which in turn is twice as important as SAR, inactive analogs and PAINS scores. However, we stress that different proposed experimental cases will require different weights of these scores in order to access the best probes. We do not think that there is a unique Global Score applicable to all chemical biology experiments and accordingly the weights for each of the scores can be personalised for individual user needs in the website resource. Note that it is unfortunately not possible to fairly compare our Global Score to the recently developed TS score for prioritisation of chemical tools for phenotypic screening as TS uses expert weighting of several databases,

including highly-curated proprietary databases for which we do not have access (Wang et al., 2016). The Global Score has the following formula (for pre-defined weights a = 8, b = 4, c = 2, d = e = f = 1):

$$Global\ Score = \frac{a \cdot Selectivity_{Score} + b \cdot Potency_{Score} + c \cdot Cell_{Score} + d \cdot SAR_{Score} + e \cdot Inactive\ Analogs_{Score} + f \cdot PAINS_{Score}}{a+b+c+d+e+f}$$

### Commercial Availability

Commercial availability is not reported as a score because we believe that this would discourage the supply of the best chemical tools and does not represent an inherent property of compounds. However, we recognise that knowing whether a chemical tool is commercially available is important for chemical tool selection and thus we provide this information in each chemical tool synopsis page. We consider a compound is commercially available if it is present in the catalogue of eMolecules (https://www.emolecules.com/) that comprises over 8 million compounds from a network of suppliers. To identify if a compound is present in the eMolecules database we use UniChem cross-references (Chambers et al., 2013).

## Development of the Probe Miner: Chemical Probes Objective Assessment Resource

We have developed an open website (http://probeminer.icr.ac.uk) using PHP, HTML and jQuery JavaScript library to enable public access to the Probe Miner resource as a framework for chemical probe prioritization using data integrated from publicly available knowledgebases.

### Target Icons

To facilitate a rapid and intuitive evaluation of chemical probe quality we have adapted the chemical probe scores to a binary representation and developed a set of icons that can be shown in colour or in grey scale depending on the chemical tool fulfilling certain criteria (Table 1). These icons are displayed in each chemical tool synopsis page (Figure 3A). Moreover, in order to facilitate a target's-eye view of chemical tool quality using these icons, the number of probes fulfilling these criteria is also displayed below the icons in each Target Overview page (Figure S3E). A description of each icon can be found in Table 1.

### Target Information Richness Score

In order to inform on the amount of information available, we develop a measure of the Information Richness for each target, not only in terms of the number of chemical compounds screened against it but also their characterization in terms of selectivity. Accordingly, for each target, every compound screened against it is counted as one unit of information. Moreover, for each compound tested against that target, each other target the compound was screened against is also counted as another unit of information. Therefore, each target has a final information value that accounts for the number of screened compounds plus the number of other targets each compound was screened against.

$$Information\ Richness_{Target\ A} = \sum_{i=1}^{Number\ of\ compounds_{Target\ A}} Number\ of\ Targets\ tested_{Compound\ i}$$

Next, each target is ranked according to their information value. The Information Richness score reports the percentile of each target in terms of ranking, being 100% for the targets with the highest information values and 0% for the targets with the lowest information values.

## Analysis of the 'Liganded' Proteome and Chemical Tools for Cancer Genes

The Potency Score (vide supra) is used to calculate how many human proteins interact with a chemical molecule with a median activity below 10,000 nM (pActivity < 5) and thus represent the currently liganded proteome. The Potency Score, Cell Score, the number of off-targets and the number of selective off-targets calculated for the Selectivity Score are subsequently used to calculate how many compound-target interactions fulfilled minimum chemical probe requirements. Only compound-target interactions with median pActivity $\leq$ 7, reported to have an affinity below 10,000 nM in at least one cell line, screened against at least one other target and at least 10-fold selective against all other targets screened are selected. Finally, the absolute number of human protein targets and chemical molecules selected is calculated. In order to compare Information Richness with chemical tool quality, information values calculated for the Target Information Richness Score (vide supra) are compared to the number of compounds fulfilling minimum chemical probe requirements for each target (Figure S1). For the analysis of minimum-quality chemical tools for cancer driver genes we extracted the chemical tools fulfilling minimum requirements from the previous analysis and annotated to the 188 cancer targets identified as potentially driving cancer in a recent pan-cancer analysis (Rubio-Perez et al., 2015).

## Analysis of Chemical Probes from The Chemical Probes Portal

All the chemical probes from The Chemical Probes Portal are downloaded from The Chemical Probes Portal website (http://www.chemicalprobes.org/browse_probes; downloaded 06/02/2017) including key information such as name, target(s) names, PubChem CID and Average Recommendation (Table S2) (Arrowsmith et al., 2015). Probes are mapped to canSAR compound IDs when possible using the provided PubChem CIDs, ChEMBLIDs or SMILES. The most potent target from the reported values is considered for the analysis and mapped to UniprotIDs via the provided gene names (Table S2). The oldest Primary Reference of the probe is also recorded and mapped to PubMed ID, journal name and publication year (Table S2). Since our assessment is performed at the single target level, we focus on the selective probes for the comparative analysis. From the 133 probes available in the resource at the

accession date, 109 were associated with no more than two targets (Table S2). Next, we selected probes that have a SAB Rating $\geq$ 3 and are thus recommended by experts following The Chemical Probes Portal guidelines (http://www.chemicalprobes.org/sab-rating-system). From the 109 selective probes, 71 are recommended by experts. From the 71 recommended probes, 46 could be mapped to publicly available medicinal chemistry databases and have affinity data for the primary target that enables the calculation of the scores. It is worth noting that many of the probes that could not be mapped were published in 2016 or 2017 and they had not yet been included in public databases such as ChEMBL. From these 46 probes, their ranking for their intended target is calculated according to our predefined Global Score (Table S2). In 30 cases (65%), the recommended probes are ranked among the top 20 by the predefined Global Score and in 17 cases (37%) the recommended probes rank among the top 5 (Figure 4). Analysis of the 15 probes that are recommended by The Chemical Probes Portal but do not rank among the top 20 by the Global Score uncovers that the main reasons for not ranking correctly are data incompleteness (mainly because key information was published in a non-indexed publication) or data inaccuracy (mainly $EC_{50}$s curated as $IC_{50}$s; Tables S2 and S3).

## DATA AND SOFTWARE AVAILABILITY

All the data calculated form the medicinal chemistry data available in canSAR (Tym et al., 2016; http://cansar.icr.ac.uk) can be downloaded from the 'Download' section of the Probe Miner website (http://probeminer.icr.ac.uk/#/download), including a list of all 355,305 human active compounds against all their human targets and their individual as well as global scores.

## ADDITIONAL RESOURCES

As part of this work, the Probe Miner website resource (http://probeminer.icr.ac.uk) has been created and made publicly available to the scientific community as a user-friendly framework to access the objective assessment method and data that we have developed. Probe Miner is a valuable resource to aid the identification of potential chemical probes, particularly when used alongside expert curation. A video tutorial is also accessible from the website (http://probeminer.icr.ac.uk/#/tutorial) to help researchers navigate the resource.

Cell Chemical Biology, Volume *25*

## Supplemental Information

# Objective, Quantitative, Data-Driven Assessment of Chemical Probes

**Albert A. Antolin, Joseph E. Tym, Angeliki Komianou, Ian Collins, Paul Workman, and Bissan Al-Lazikani**

## Supplemental Information

# Objective, Quantitative, Data-Driven Assessment of Chemical Probes

Albert A. Antolin,[1,2] Joe E. Tym,[1] Angeliki Komianou,[1] Ian Collins,[1] Paul Workman[1] & Bissan Al-Lazikani[1,2]

[1]Cancer Research UK Cancer Therapeutics Unit, Division of Cancer Therapeutics and [2]Department of Data Science, The Institute of Cancer Research, London, UK.

# Supplemental Figures


| Supplementary | Title | Page # |
|---|---|---|
| Figure S1 | No correlation existing between Information Richness and number of minimum quality chemical tools per each target. | 3 |
| Figure S2 | Probing activating cancer driver targets. | 4 |
| Figure S3 | Flow diagram illustrating the logic for the calculation of the scores. | 5 |
| Figure S4 | Identification and correction of a curation error in ChEMBL affecting chemical probe CCT241533 | 6 |
| Figure S5 | Theoretical examples | 7 |
| Figure S6 | Most compound-target interactions are not affected by a very broad distribution of affinity values. | 8 |

**Figure S1. No correlation existing between Information Richness and number of minimum quality chemical tools per each target, related to Figure 1 and STAR Methods**. The red line represents the failed linear regression of the data that has an $R^2$ of 0.1093.

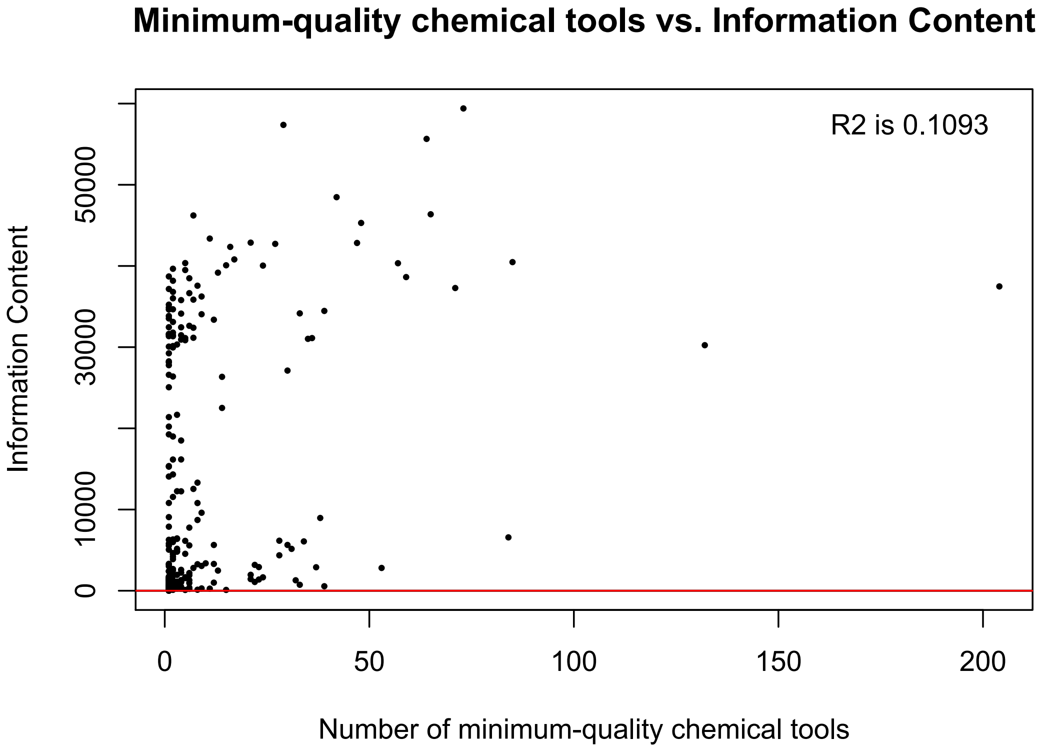

**Figure S2. Probing activating cancer driver targets. On the left, pie chart representing the number of cancer driver genes with minimum-quality chemical tools, related to Figure 1.** On the right, protein-protein interaction network of the 25 cancer driver genes that have minimum-quality chemical probes obtained from canSAR (Tym et al., 2016). Node size is proportional to the number of compounds tested for the target and shading represents the number of minimum-quality chemical probes, being dark blue the largest number of probes. Edges are coloured depending on the type of protein-protein interaction. Black represents direct binding, cyan represents phosphorylation with arrow showing direction, and magenta represent crystallographycally resolved interactions.

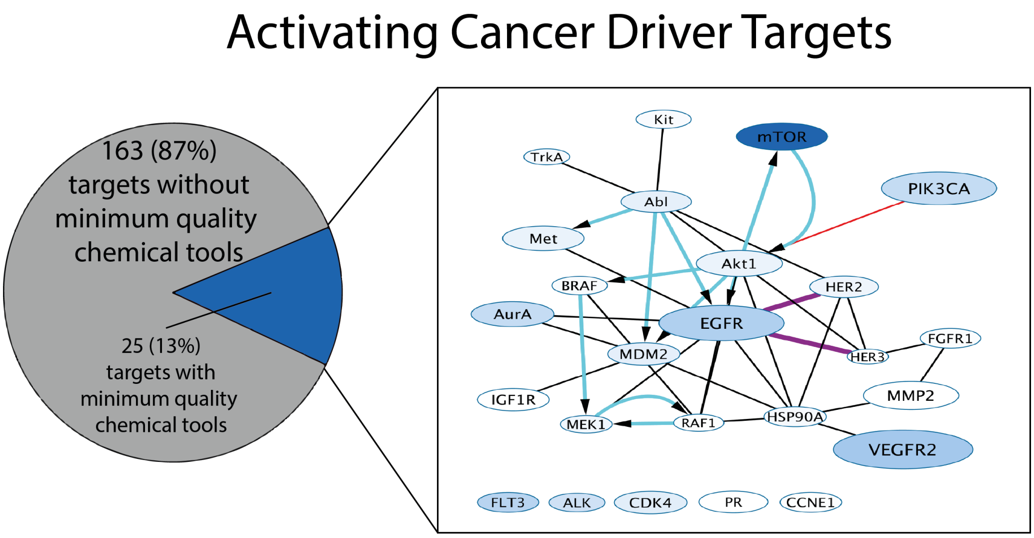

**Figure S3. Flow diagram illustrating the logic for the calculation of the scores, related to STAR Methods.**

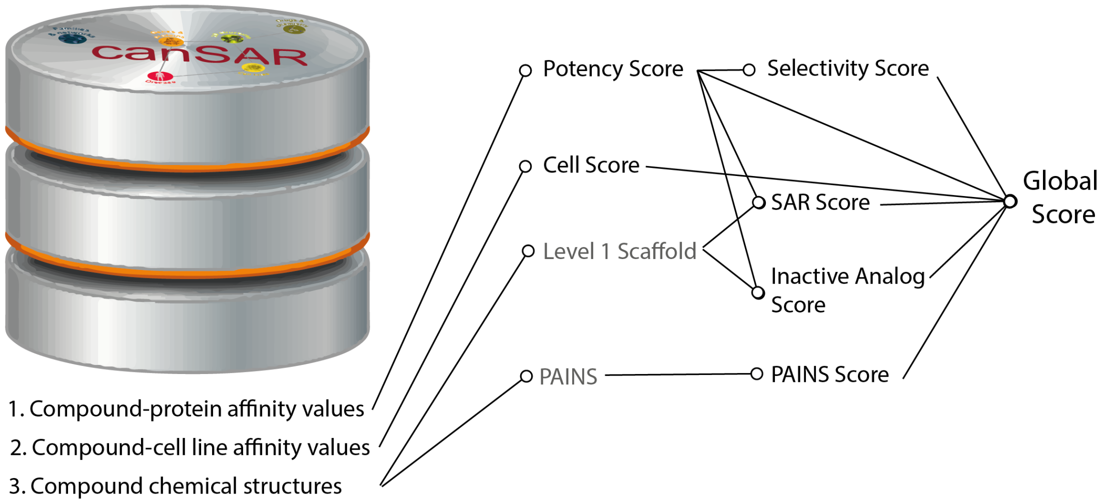

**Figure S4. Identification and correction of a curation error in ChEMBL22 affecting chemical probe CCT241533, related to Figure 4.** While analysing the reasons for not ranking among the top 20 chemical tools 15 of the chemical probes recommended by The Chemical Probes Portal (Supplementary Table 1-2), we identified that the CHEK2 $IC_{50}$ for CCT241533 had been wrongly curated in ChEMBL22 (CHEMBL1236782) and a value of 30 nM had been given instead of 3 nM as in the original publication. This lower affinity made the probe appear as not 10-fold selective and therefore the Selectivity Score was very low and the Potency Score was also lower than deserved. We have thus corrected this value in canSAR and informed ChEMBL of the error so it can be corrected in future versions of the ChEMBL database. The probe now scores 3rd in our resource when ranking CHEK2 chemical tools by the predefined Global Score. This example illustrates that public databases are not exempt of errors that need to be corrected to make best use of this resource.

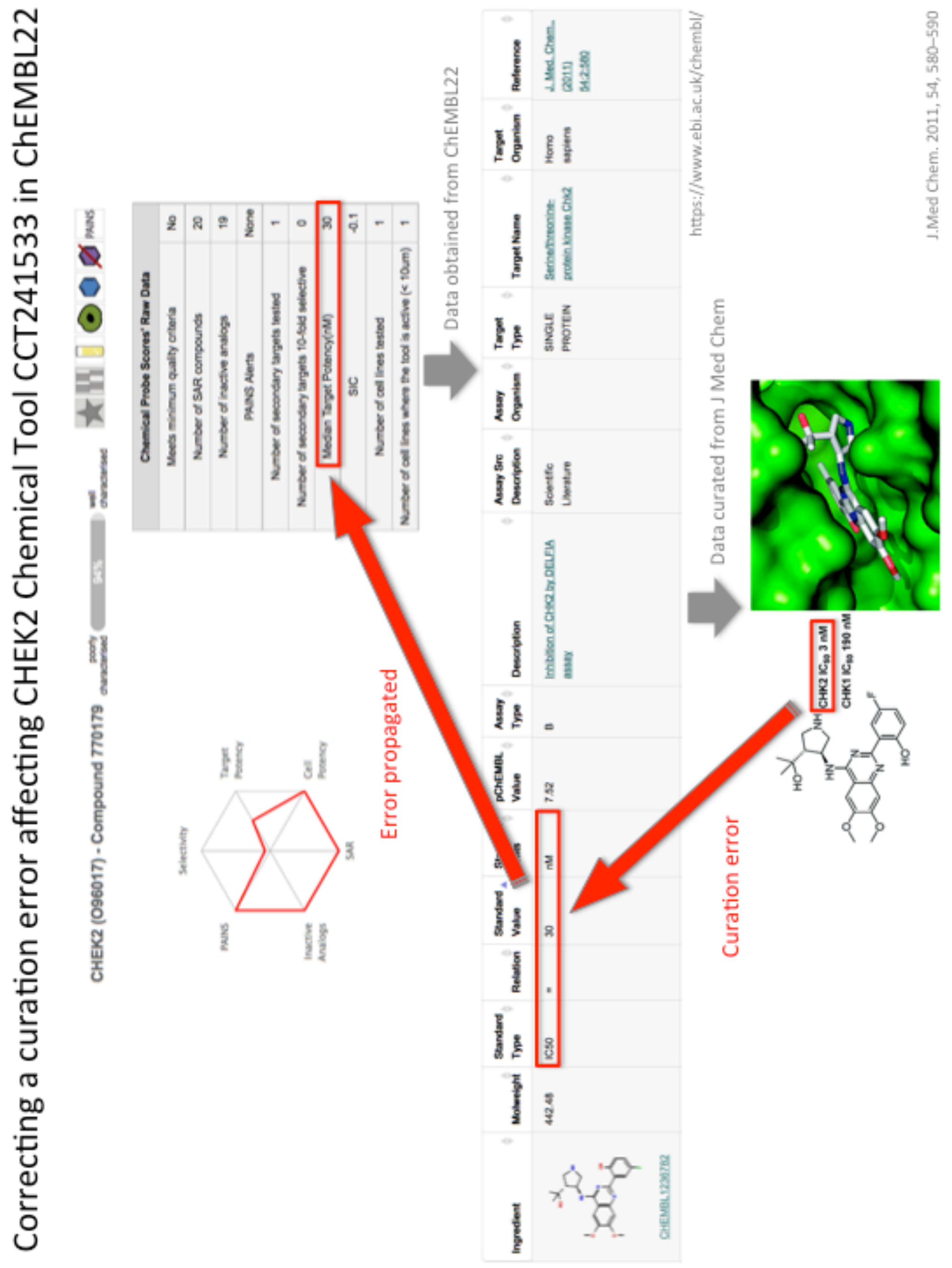

**Figure S5. Theoretical examples, related to STAR Methods.** (A) Example showing how the differences in selectivity knowledge challenge the comparison of molecules screened against a very different number of targets. (B) Theoretical example illustrating the calculation of the number of off-targets, the differentiation between selective and unselective off-targets and the calculation of first factor of the Selectivity Score. (C) Calculation of the SIC and the Second Factor. (D) Comparing the SIC and Second Factor measures for compounds screened against a very different number of targets.

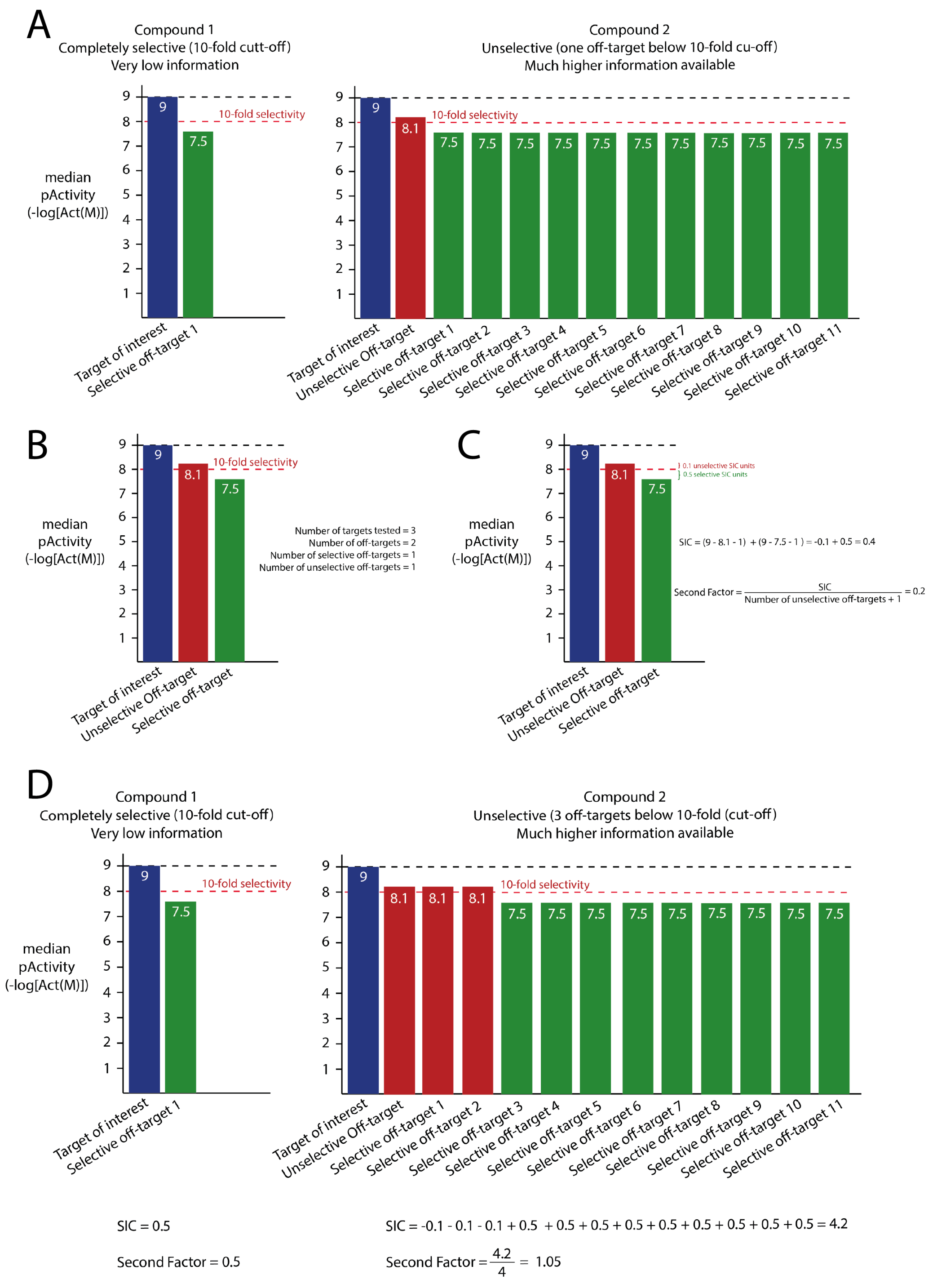

**Figure S6. Most compound-target pairs are not affected by a very broad distribution of affinity values, related to STAR Methods**. To identify compound-target pairs with broad distribution we calculated the difference between the highest and the lowest values when adding or subtracting the Median Absolute Deviation (MAD) to the Median in a logarithmic scale. As it can be observed in the histogram, for the vast majority of compound-target pairs (96.4%) there is less than 10-fold difference (1 log unit) between these extreme values, thus supporting the use of the median in most cases.

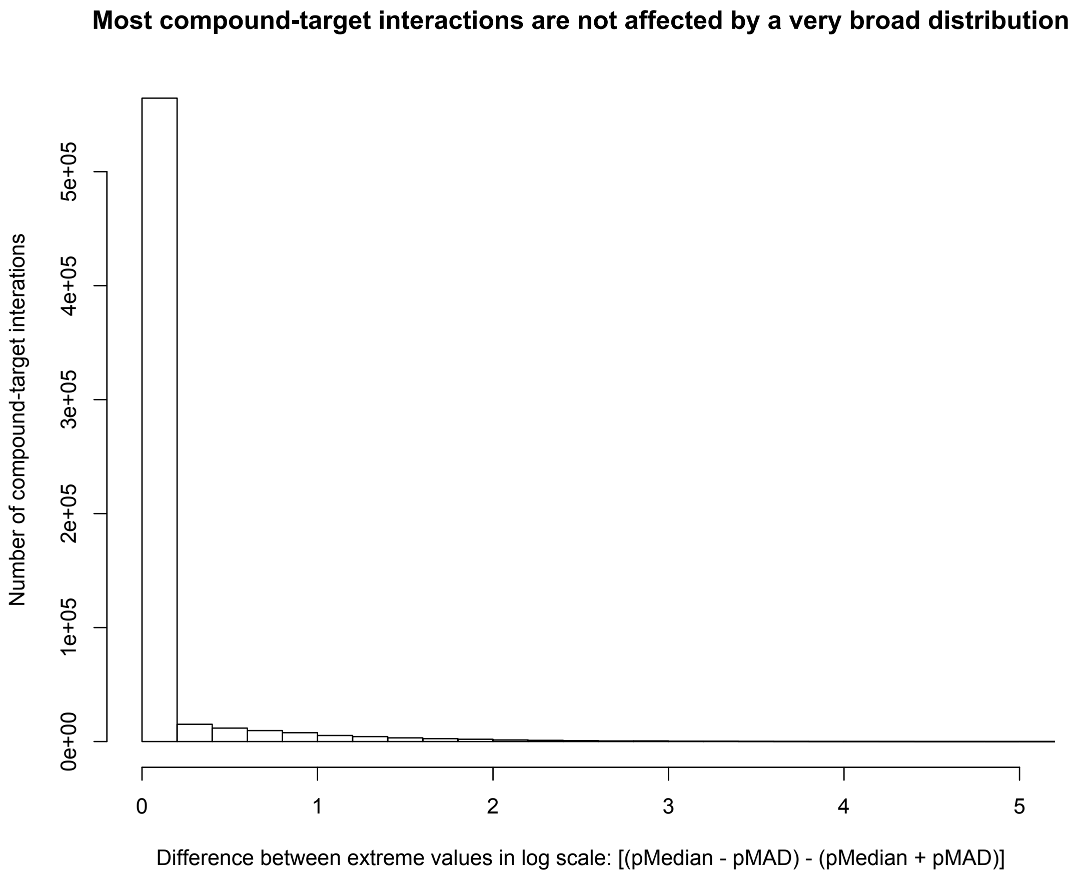

Supplement: Document S2. Article plus Supplemental Information [file mmc6.pdf]
